# Protective Effect and Mechanism of Aspirin Eugenol Ester on Lipopolysaccharide-Induced Intestinal Barrier Injury

**DOI:** 10.3390/ijms242417434

**Published:** 2023-12-13

**Authors:** Qi Tao, Xi-Wang Liu, Zhen-Dong Zhang, Ning Ma, Xiao-Rong Lu, Wen-Bo Ge, Jian-Yong Li, Ya-Jun Yang

**Affiliations:** 1 Key Lab of New Animal Drug Project of Gansu Province, Key Lab of Veterinary Pharmaceutical Development of Ministry of Agriculture and Rural Affairs, Lanzhou Institute of Husbandry and Pharmaceutical Sciences of CAAS, Lanzhou 730050, China; taoqi19951224@163.com (Q.T.); xiwangliu@126.com (X.-W.L.); 13027721013@163.com (Z.-D.Z.); luxr1993@163.com (X.-R.L.); gewb1993@163.cm (W.-B.G.); 2 College of Veterinary Medicine, Hebei Agricultural University, Baoding 071001, China; maning9618@163.com

**Keywords:** aspirin eugenol ester (AEE), intestinal barrier, tight junction, inflammation, gut microbiota

## Abstract

Intestinal inflammation is a complex and recurrent inflammatory disease. Pharmacological and pharmacodynamic experiments showed that aspirin eugenol ester (AEE) has good anti-inflammatory, antipyretic, and analgesic effects. However, the role of AEE in regulating intestinal inflammation has not been explored. This study aimed to investigate whether AEE could have a protective effect on LPS-induced intestinal inflammation and thus help to alleviate the damage to the intestinal barrier. This was assessed with an inflammation model in Caco-2 cells and in rats induced with LPS. The expression of inflammatory mediators, intestinal epithelial barrier-related proteins, and redox-related signals was analyzed using an enzyme-linked immunosorbent assay (ELISA), Western blotting, immunofluorescence staining, and RT-qPCR. Intestinal damage was assessed by histopathological examination. Changes in rat gut microbiota and their functions were detected by the gut microbial metagenome. AEE significantly reduced LPS-induced pro-inflammatory cytokine levels (*p* < 0.05) and oxidative stress levels in Caco-2 cells and rats. Compared with the LPS group, AEE could increase the relative expression of Occludin, Claudin-1, and zonula occludens-1 (ZO-1) and decrease the relative expression of kappa-B (NF-κB) and matrix metalloproteinase-9. AEE could significantly improve weight loss, diarrhea, reduced intestinal muscle thickness, and intestinal villi damage in rats. Metagenome results showed that AEE could regulate the homeostasis of the gut flora and alter the relative abundance of *Firmicutes* and *Bacteroidetes*. Flora enrichment analysis indicated that the regulation of gut flora with AEE may be related to the regulation of glucose metabolism and energy metabolism. AEE could have positive effects on intestinal inflammation-related diseases.

## 1. Introduction

Enteritis is defined as inflammation of the superficial and deeper tissues of the intestinal tract, resulting in various degrees of edema, congestion, hemorrhage, ulceration, suppuration, and necrosis due to stimulation by pathogenic factors [1,2]. Intestinal tract mucosal epithelial cells can differentiate into successive monolayers that form an intestinal epithelial barrier; the intestinal epithelial barrier can prevent harmful microorganisms and bacterial endotoxins from coming into contact with the intestinal epithelial cells and thereby destroying them [3]. Therefore, the intestinal epithelial barrier plays a key role in fighting microbial-induced gastrointestinal diseases. The intestinal mucosal epithelial cell junctions are a major component of the intact intestinal epithelial barrier and consist mainly of tight junction (TJ) proteins, cell membranes, and related proteins such as zonula occludens (ZO), Claudins, and Occludin, whose expressions play a crucial role in maintaining intestinal barrier function [4,5]. It was reported that nuclear factor kappa-B (NF-κB) is an important intracellular signal transduction and transcription factor, which regulates the expression and assembly of TJ proteins and promotes the contraction of actin and TJ [6]. Several studies have also pointed out the pathogenic role of matrix metalloproteinase-9 (MMP-9) in animal models of inflammatory bowel disease [7,8]. The intestine is also an important organ with a high level of immunity. When the organism is invaded by microorganisms, a variety of cytokines are rapidly secreted in a short time, which exceeds the range of the organism’s immune regulation, causing a local inflammatory response and damaging the organism’s tissues and organs [9].

Intestinal flora is considered the “second brain” or “gut brain” of the body [10]. In normal physiological conditions, the intestinal flora is involved in a variety of intestinal metabolisms, maintains the integrity of the intestinal mucosa, eliminates pathogenic microorganisms, and influences the host immune system. When inflammation occurs in the intestines, the gut flora can become severely dysbiotic [11]. Usually, intestinal flora dysbiosis leads to metabolic dysfunction and immune disorders [12]. Therefore, alleviating intestinal flora dysbiosis may be beneficial to prevent or mitigate the occurrence of intestinal inflammatory response.

Lipopolysaccharide (LPS) is one of the important components of the cell wall of Gram-negative bacteria. LPS consists of Q side chains, core polysaccharides, and lipid-like A. Lipid-like A is the main virulence center and biologically active part of LPS, which is highly conserved and non-species-specific. The toxic effects of LPS produced by different strains of bacteria after infection are approximately the same [13]. LPS can lead to increased intestinal permeability and intestinal mucosal damage, which play a key role in mediating the intestinal inflammatory response [14]. Therefore, LPS injury models are widely used in studies concerning the intestinal barrier.

AEE (aspirin eugenol ester) is a novel medicinal compound synthesized by esterification of aspirin with eugenol using the twin drug principle. It has various pharmacological activities. In previous studies, the anti-inflammatory, anti-oxidative stress effects of AEE have been confirmed. For example, AEE reduced Paraquat-induced hepatotoxicity by inhibiting oxidative stress and maintaining mitochondrial function [15]. AEE protected ASK1 (apoptosis signal-regulating kinase 1) pathways by mediating vascular endothelial cells (VECs) from oxidative damage [16]. AEE pretreatment could significantly enhance the cellular superoxide dismutase and glutathione peroxidase activities in VECs [17]. Another experimental result showed that AEE pretreatment could improve the adverse effects of VECs caused by H_2_O_2_ [18]. The further study showed that AEE protects VECs from oxidative damage by regulating NOS (nitric oxide synthase) and Nrf2 (nuclear factor erythroid-derived 2-like 2) signaling pathways [19]. 5-aminosalicylic acid (5-ASA) is widely used in clinical practice for the treatment of colitis, especially ulcerative colitis [20]. 5-ASA could improve dextran sulphate sodium (DSS)-induced colitis in mice by regulating intestinal microbiota and bile acid metabolism [21]. Therefore, it is speculated that AEE may also have some preventive and palliative value in intestinal inflammation. Based on the previous study results, this experiment was designed for the oral administration of AEE to LPS-induced rats to evaluate whether AEE has a protective effect on LPS-induced intestinal inflammation, which may help to reduce the damage to the intestinal barrier. This will provide theoretical support and ideas for the application of AEE in intestinal inflammation.

## 2. Results

### 2.1. AEE Alleviated LPS-Induced Cytotoxicity

Drug cytotoxicity was assessed by cell viability. Compared with the control group, there was no change in the survival rate of Caco-2 cells treated with 0–100 μg·mL^−1^ LPS for 24 h and/or 0–128 μM AEE for 24 h (Figure S1A–C, *p* > 0.05). AEE was analyzed for its protective effect against LPS (100 μg·mL^−1^)-induced cytotoxicity by measuring LDH leakage. Caco-2 cells were pretreated with AEE (8, 32, 64 μM) for 24 h and then treated with LPS for 24 h. The results showed that AEE significantly reduced LPS-induced LDH release (*p* < 0.05, Figure S1D).

### 2.2. AEE Alleviated LPS-Induced Oxidative Stress

**Figure 1:**
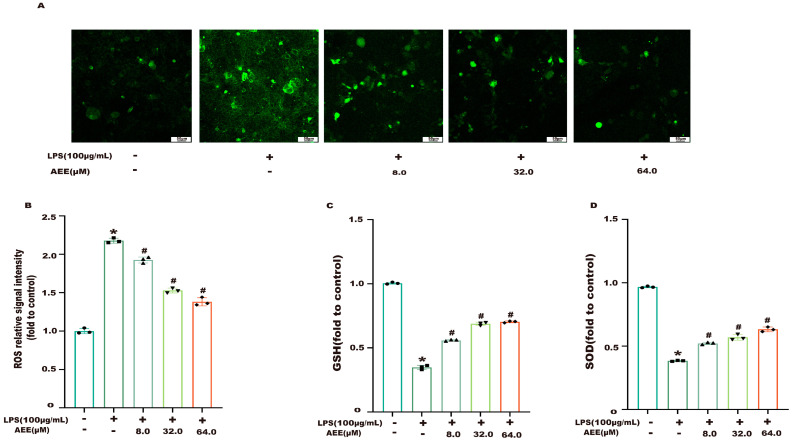
Effect of AEE on LPS-induced oxidative stress. Caco-2 cells were pretreated with AEE (8, 32, 64 μM) for 24 h and then treated with LPS (100 μg·mL^−1^) for 24 h. (**A**) Representative fluorescent images of ROS. (**B**) ROS. (**C**) GSH. (**D**) SOD. Values are presented as the means ± SD where applicable (*n* = 6). * *p* < 0.05 compared to control group, ^#^ *p* < 0.05 compared to LPS group. One-way ANOVA followed by Duncan’s multiple comparisons.

The results of AEE alleviating LPS-induced oxidative stress are shown in Figure 1A,B. Compared with the control group, the ROS level of Caco-2 cells was significantly increased in the LPS group (*p* < 0.05). Compared with the LPS group, the ROS levels of Caco-2 cells in the AEE group were significantly decreased in a dose-dependent manner (*p* < 0.05). On the other hand, as shown in Figure 1C,D, the levels of GSH and SOD were significantly decreased in the LPS group compared with control group (*p* < 0.05). Compared with the LPS group, there was a significant increase in the levels of GSH and SOD in the AEE group (*p* < 0.05), which indicated that the AEE pretreatment could alleviate the LPS-induced oxidative stress to some extent.

### 2.3. AEE Alleviated LPS-Induced Inflammatory Responses

**Figure 2:**
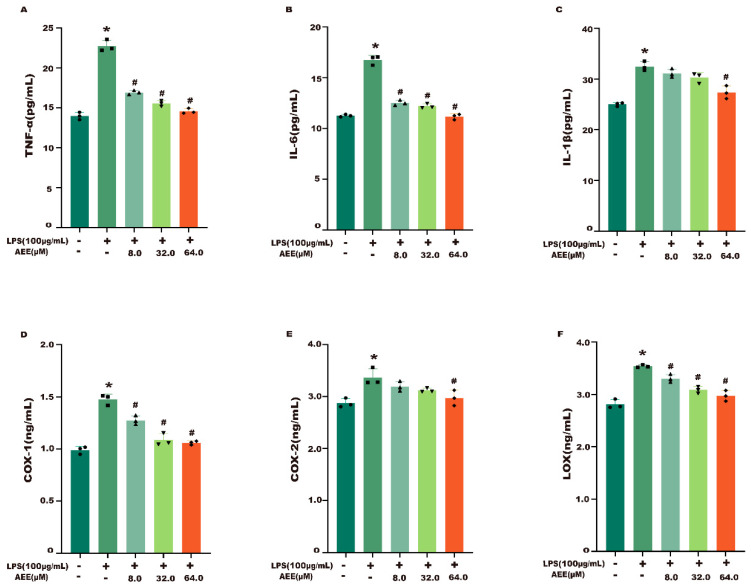
Effect of AEE on LPS-induced relevant inflammatory indicators. Caco-2 cells were pretreated with AEE (8, 32, 64 μM) for 24 h and then treated with LPS (100 μg·mL^−1^) for 24 h. (**A**) TNF-α content, (**B**) IL-6 content, (**C**) IL-1β content, (**D**) COX-1 content, (**E**) COX-2 content, and (**F**) LOX content in the supernatant of Caco-2 cells. Values are presented as the means ± SD where applicable (*n* = 6). * *p* < 0.05 compared to control group, ^#^ *p* < 0.05 compared to LPS group. One-way ANOVA followed by Duncan’s multiple comparisons.

The results in Figure 2 showed that the levels of TNF-α, IL-6, IL-1β, COX-1, COX-2, and LOX were higher in the LPS group than in the control group (*p* < 0.05). Compared with the LPS group, AEE pretreatment significantly inhibited the LPS-induced upregulation of these indicators (*p* < 0.05). The data indicated that AEE inhibition of the LPS-induced inflammatory response might be accomplished by inhibiting these indicators.

### 2.4. Effects of AEE on the Morphology of LPS-Induced Caco-2 Cells

**Figure 3:**
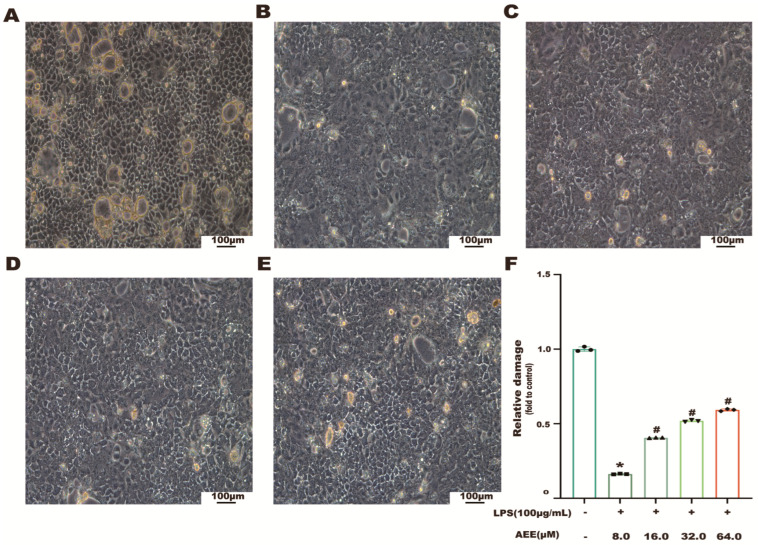
Effect of AEE on LPS-induced morphology of Caco-2 cells observed by inverted light microscopy. Caco-2 cells were pretreated with AEE (8, 32, 64 μM) for 24 h and then treated with LPS (100 μg·mL^−1^) for 24 h. (**A**–**E**) MEM (blank), LPS (100 μg·mL^−1^), AEE (8 μM) + LPS (100 μg·mL^−1^), AEE (32 μM) + LPS (100 μg·mL^−1^), and AEE (64 μM) + LPS (100 μg·mL^−1^) combinations. (**F**) Relative damage was calculated by Image J software. Values are presented as the means ± SD where applicable (*n* = 3). * *p* < 0.05 compared to control group, ^#^ *p* < 0.05 compared to LPS group. One-way ANOVA followed by Duncan’s multiple comparisons.

The results are shown in Figure 3A–E. The state of the Caco-2 cells in the control group was uniform and dense, with TJs between cells. Compared with the control group, the Caco-2 cell junctions in the LPS group were broken and the cytoskeleton was lost. Compared with the LPS group, the junctions between cells in the AEE group were all protected to some extent and showed a dose–effect relationship. It suggested that AEE could alleviate LPS-induced damage to Cao-2 cell monolayers.

### 2.5. AEE Alleviated LPS-Induced Caco-2 Cell Monolayer Damage

**Figure 4:**
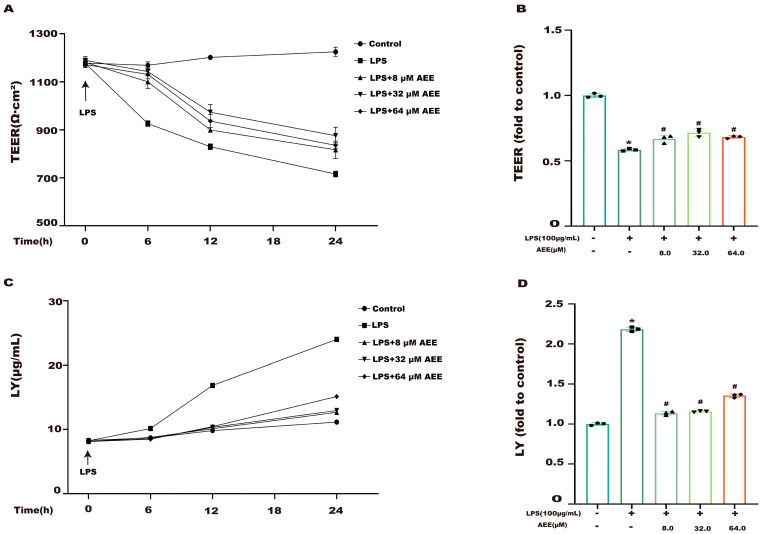
Effect of AEE on LPS-induced monolayer Caco-2 cell integrity. Caco-2 cells were pretreated with AEE (8, 32, 64 μM) for 24 h and then treated with LPS (100 μg·mL^−1^) for 24 h. (**A**) TEER of Caco-2 cells with time, (**B**) final value of TEER, (**C**) LY permeability of Caco-2 cells with time, (**D**) final value of LY permeability. Values are presented as the means ± SD where applicable (*n* = 3). * *p* < 0.05 compared to control group, ^#^ *p* < 0.05 compared to LPS group. One-way ANOVA followed by Duncan’s multiple comparisons.

To evaluate the improvement effect of AEE on LPS-induced Caco-2 cell monolayer damage, this study measured TEER and LY permeability. The results are shown in Figure 4A,B. Compared with the control group, the TEER decreased significantly in the LPS group (*p* < 0.05). The TEER increased significantly in the AEE group compared to the LPS group (*p* < 0.05). In addition, as shown in Figure 4C,D, AEE could significantly inhibit the increase in LY permeability caused by LPS (*p* < 0.05). It indicated that AEE could improve the damage of the Caco-2 cell monolayer caused by LPS.

### 2.6. AEE Inhibited LPS-Induced Downregulation of TJ Proteins

**Figure 5:**
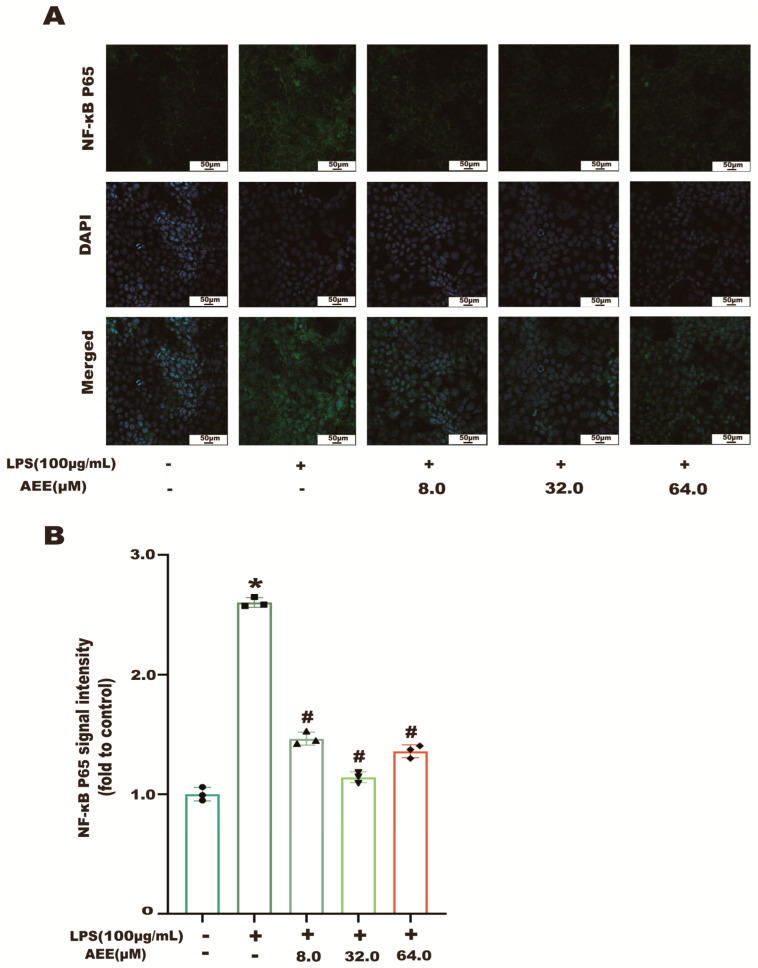
Effect of AEE on LPS-induced downregulation of TJ proteins. Caco-2 cells were pretreated with AEE (8, 32, 64 μM) for 24 h and then treated with LPS (100 μg·mL^−1^) for 24 h. (**A**) Immunofluorescence staining of TJ protein ZO-1 in Caco-2 cells. (**B**) Relative fluorescence intensity of ZO-1 was calculated by Image J software 1.8.0. Values are presented as the means ± SD where applicable (*n* = 3). * *p* < 0.05 compared to control group, ^#^ *p* < 0.05 compared to LPS group. One-way ANOVA followed by Duncan’s multiple comparisons.

**Figure 6:**
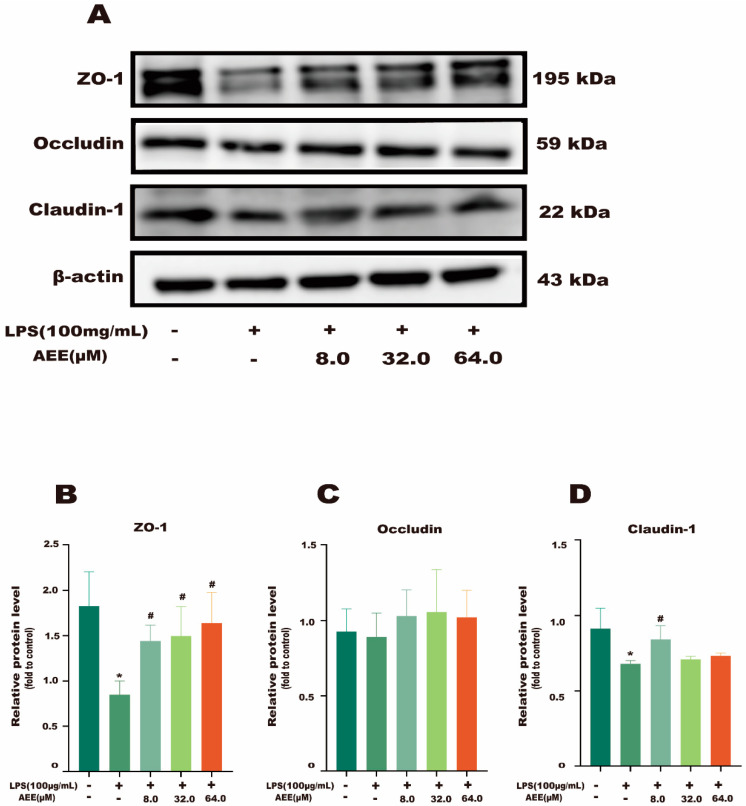
Effect of AEE on LPS-induced downregulation of TJ proteins. Caco-2 cells were pretreated with AEE (8, 32, 64 μM) for 24 h and then treated with LPS (100 μg·mL^−1^) for 24 h. (**A**) Protein levels of ZO-1, Occludin, and Claudin-1 were detected by Western blot. (**B**–**D**) Relative protein expressions of ZO-1, Occludin, and Claudin-1 were calculated by Image J software 1.8.0. Values are presented as the means ± SD where applicable (*n* = 3). * *p* < 0.05 compared to control group, ^#^ *p* < 0.05 compared to LPS group. One-way ANOVA followed by Duncan’s multiple comparisons.

To further investigate the protective effect of AEE on the Caco-2 cell monolayer, this experiment used cellular immunofluorescence to observe the fluorescence intensity of the TJ proteins (ZO-1, Occludin, and Claudin-1) on the cell membrane. The results in Figure 5 and Figures S2 and S3 show that the fluorescence signals of TJ proteins (ZO-1, Occludin, and Claudin-1) in the control group were distributed uniformly at the cell membrane. Compared with the control group, the fluorescence signals of TJ proteins (ZO-1, Occludin, and Claudin-1) in the LPS group were distributed unevenly under the cell membrane, the fluorescence intensity decreased, the joints broke obviously, and LPS disrupted the TJs of Caco-2 cell monolayer. Compared with the LPS group, the AEE group had improved fluorescence signal distribution and joints. In addition, this experiment also used a Western blot assay for detecting the expression of TJ proteins. The results shown in Figure 6A–D show that the expression of TJ proteins (ZO-1, Occludin, and Claudin-1) was significantly lower in the LPS group compared with the control group (*p* < 0.05). Compared with the LPS group, the expression of TJ proteins (ZO-1, Occludin, and Claudin-1) was significantly higher in the AEE group (*p* < 0.05). This suggests that AEE pretreatment could inhibit the downregulation of Caco-2 monolayer TJ proteins caused by LPS.

### 2.7. AEE Inhibited LPS-Induced NF-κB and MMP-9 Activation

**Figure 7:**
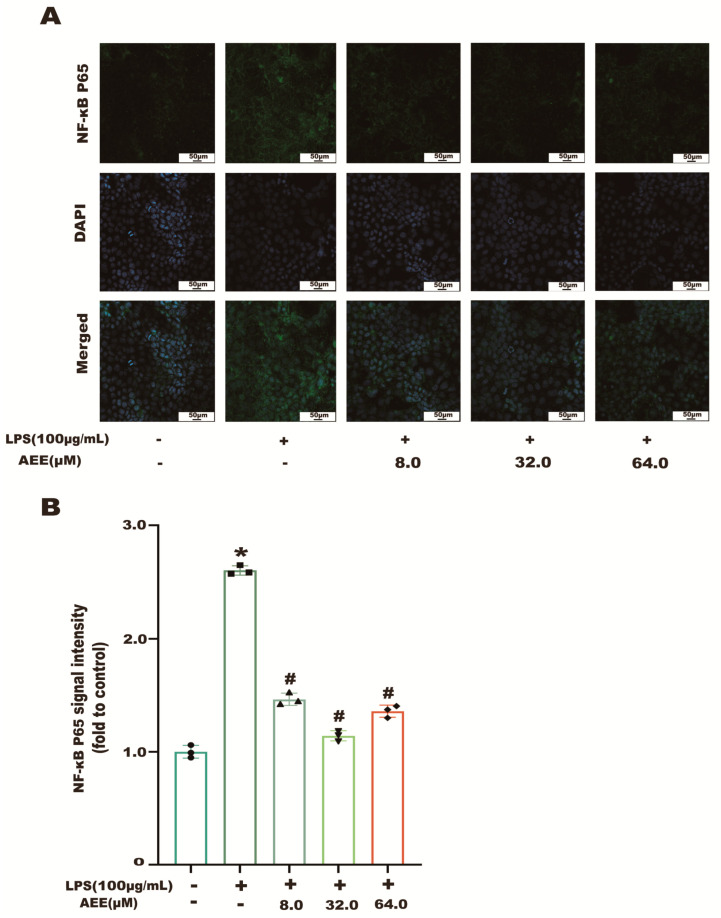
Effect of AEE on NF-κB. Caco-2 cells were pretreated with AEE (8, 32, 64 μM) for 24 h and then treated with LPS (100 μg·mL^−1^) for 24 h. (**A**) Immunofluorescence staining of NF-κB in Caco-2 cells. (**B**) Relative fluorescence intensity of NF-κB was calculated by Image J software 1.8.0. Values are presented as the means ± SD where applicable (*n* = 3). * *p* < 0.05 compared to control group, ^#^ *p* < 0.05 compared to LPS group. One-way ANOVA followed by Duncan’s multiple comparisons.

**Figure 8:**
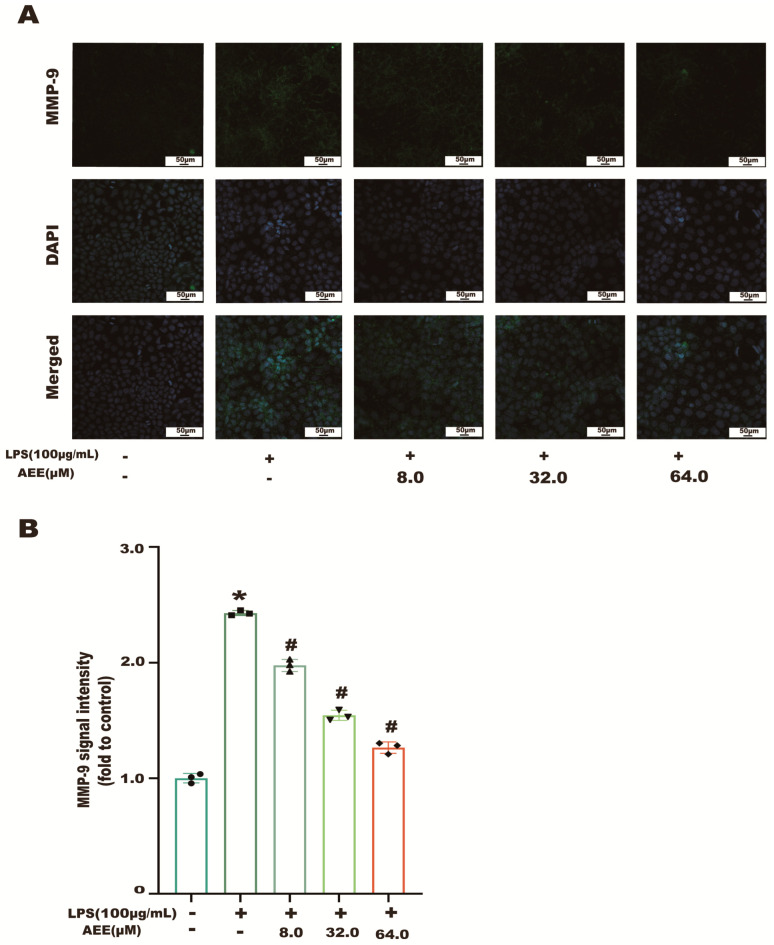
Effect of AEE on MMP-9. Caco-2 cells were pretreated with AEE (8, 32, 64 μM) for 24 h and then treated with LPS (100 μg·mL^−1^) for 24 h. (**A**) Immunofluorescence staining of MMP-9 in Caco-2 cells. (**B**) Relative fluorescence intensity of MMP-9 was calculated by Image J software 1.8.0. Values are presented as the means ± SD where applicable (*n* = 3). * *p* < 0.05 compared to control group, ^#^ *p* < 0.05 compared to LPS group. One-way ANOVA followed by Duncan’s multiple comparisons.

**Figure 9:**
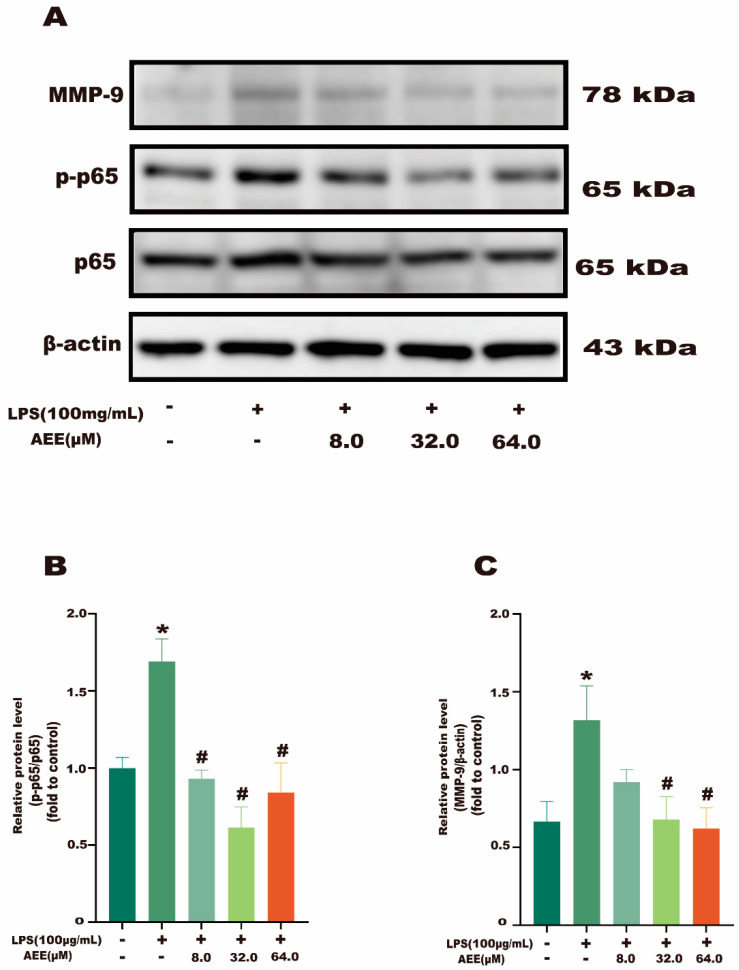
Effect of AEE on MMP-9 and NF-κB. Caco-2 cells were pretreated with AEE (8, 32, 64 μM) for 24 h and then treated with LPS (100 μg·mL^−1^) for 24 h. (**A**) Protein levels of MMP-9 and NF-κB were detected by Western blot. (**B**,**C**) Relative protein expressions of MMP-9 and NF-κB were calculated by Image J software1.8.0. Values are presented as the means ± SD where applicable (*n* = 3). * *p* < 0.05 compared to control group, ^#^ *p* < 0.05 compared to LPS group. One-way ANOVA followed by Duncan’s multiple comparisons.

The results in Figure 7A showed that the NF-κB p65 green fluorescence signal from the control group was weak and distributed in the cytoplasm. Compared with the control group, the NF-κB p65 green fluorescence signal was significantly enhanced in the LPS group (*p* < 0.05), as well as the transfer of NF-κB p65 green fluorescence from the cytoplasm to the nucleus, showing cytoplasmic and nuclear co-staining. Compared with the LPS group, the fluorescence intensity of NF-κB p65 in the nucleus was significantly decreased in the AEE group (*p* < 0.05). This indicates that AEE pretreatment could inhibit the transfer of NF-κB p65 from the cytoplasm to the nucleus to some extent. On the other hand, as shown in Figure 8B, compared with the control group, the green fluorescence signal of MMP-9 was significantly enhanced in the LPS group (*p* < 0.05). The fluorescence intensity of MMP-9 in the cells of the AEE group was significantly decreased compared with the LPS group (*p* < 0.05). Moreover, this experiment also used the protein blotting assay for detecting the expression of NF-κB p65 and MMP-9 proteins. The results in Figure 9A–C show that p65 expression was significantly increased in the cell nuclei of the LPS group compared with the control group (*p* < 0.05), and AEE pretreatment significantly inhibited the above responses. AEE pretreatment also significantly inhibited the expression of MMP-9 in LPS-induced Caco-2 cells (*p* < 0.05). These results suggest that AEE could alleviate the TJ damage due to LPS by inhibiting the expression of NF-κB and MMP-9 proteins.

### 2.8. Metabolomic Analysis of LPS-Induced Caco-2 Cells

#### 2.8.1. Metabolite Analysis of Cell Lysates

The metabolite data from the cell lysate samples of the three experimental groups were further analyzed using principal component analysis (PCA). As shown in Figure S4A,B,G,H, the metabolites of the three experimental groups changed and had large differences. To reduce the influence of irrelevant factors on the analytical results and to improve the separation and identification of metabolites, this experiment also used orthogonal partial least squares discriminant analysis (OPLS-DA) on the three experimental groups for modeling and analysis. As shown in Figure S4C–F,I–K, there was a clear separation between the LPS group and the other groups, with no overlap of positive or negative patterns. Potential differential metabolites were selected in the OPLS-DA model using VIP (VIP > 1) and *t*-test *p* < 0.05, and differential metabolites were analyzed using target MS/MS scans. Secondary characteristic ion fragments of the differential metabolites were obtained for comparison in relevant databases. As shown in Table S4, the analysis of cell lysate samples eventually screened 24 differential metabolites, including quinmerac, arborinine, uridine 5-(trihydrogen diphosphate), glutamate conjugated chenodeoxycholic acid, quercetin pentamethyl ether, jervine, buxtamine, peiminine, thymine, isoguvacine, caffeoylcholine, fumaric acid, salicyclic acid, L-cystine, UDP-D-glucuronic acid, cytarabine, 6-methoxyflavonol, butylparaben, fisetin, phosphatidylserine 18, triptophenolide, taurolithocholic acid, labetalol, and aurapten. Compared with the LPS group, the levels of these differential metabolites were restored after pretreatment with AEE. This indicates that AEE could improve the metabolic changes induced by LPS in Caco-2 cells.

#### 2.8.2. Metabolic Pathway Analysis

To investigate the effect of AEE on the metabolic pathway of LPS-induced Caco-2 cells, the metabolic pathway was analyzed using MetaboAnalyst 5.0. As shown in Figure S5, there were mainly 13 metabolic pathways: tyrosine metabolism; ascorbate and aldarate metabolism; ubiquinone and other terpenoid-quinone; arginine biosynthesis; glycerolipid metabolism; pentose and glucuronate interconversions; citrate cycle; pyruvate metabolism; galactose metabolism; alanine, aspartate, and glutamate metabolism; cysteine and methionine; amino sugar and nucleotide sugar metabolism; and pyrimidine metabolism. After LPS treatment, LPS mainly leads to problems in oxidative stress, energy metabolism, and glucose metabolism in Caco-2 cells, but AEE could mitigate these to some extent.

### 2.9. AEE Pretreatment Alleviated LPS-Induced Colitis in Rats

**Figure 10:**
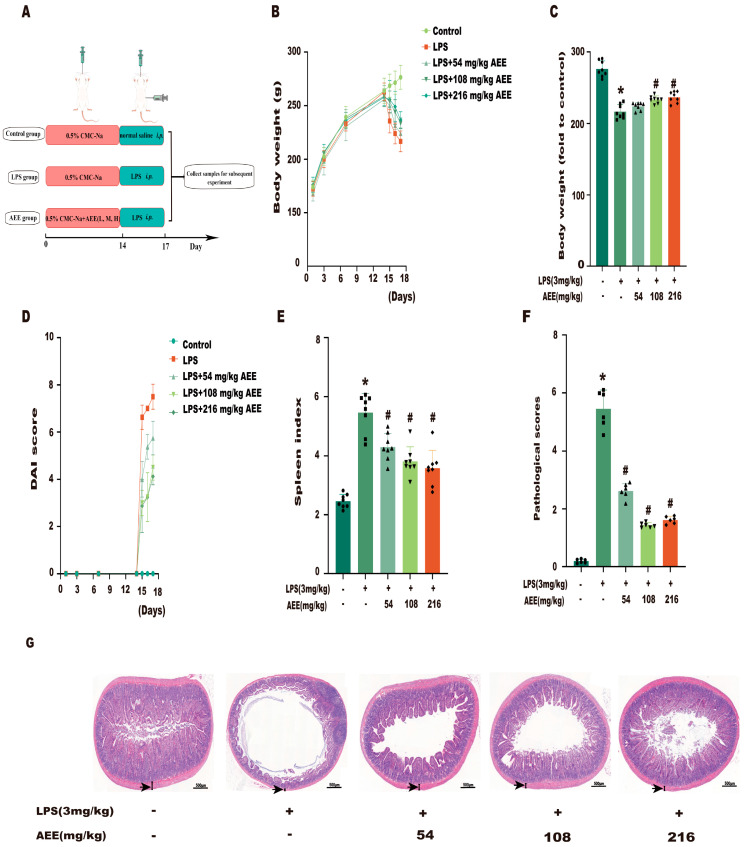
Effect of AEE on LPS-induced colitis in rats. (**A**) Experimental design. (**B**) Body weight changes in rats. (**C**) Final value of body weight. (**D**) Disease activity index score. (**E**) The effect of AEE on the splenic index of LPS-induced colitis rats. (**F**) Pathological scores of colonic tissue sections. (**G**) Effect of AEE on pathological symptoms of LPS-induced colitis in rats, arrows point to the thickness of the muscle layer. Values are presented as the means ± SD where applicable (*n* = 8). * *p* < 0.05 compared to control group, ^#^ *p* < 0.05 compared to LPS group. One-way ANOVA followed by Duncan’s multiple comparisons.

Firstly, this experiment established the LPS-induced colitis model in rats and then evaluated whether AEE alleviated the symptoms of LPS-induced colitis in rats, and the animal experimental protocol is shown in Figure 10A. As shown in Figure 10B,C, compared with the control group, the rats in the LPS group continued to lose body weight after LPS treatment (*p* < 0.05). Compared with the LPS group, the rats in the AEE group lost less body weight, which indicated that AEE could inhibit this downward trend (*p* < 0.05). As shown in Figure 10D, compared to the control group, after 3 days of LPS treatment, rats exhibited typical signs of colitis, including diarrhea, blood in the stool, and weight loss. These symptoms were indicated via higher DAI scores. Compared to the LPS group, rats in the AEE group had a significantly lower DAI score (*p* < 0.05). In addition, the results showed in Figure 10E that the splenic index was significantly increased in the LPS group compared with control group (*p* < 0.05). Compared with the LPS group, the splenic index in the AEE group was significantly decreased (*p* < 0.05), which indicated that AEE could inhibit the increase in the splenic index. Next, HE staining of colonic tissues was performed for histopathological observation. As shown in Figure 10F–G, compared with the control group, the colon tissue in the LPS group had inflammatory cell infiltration, damage to the choriocapillaris, and a decrease in the thickness of the muscle layer. Compared with the LPS group, AEE alleviated the damage to the choriocapillaris and inhibited the reduction in the thickness of the muscle layer in the colon tissue of rats caused by LPS, which indicated that AEE could alleviate the LPS-induced histopathological damage in the colon to some extent. These results indicate that AEE could improve the symptoms associated with LPS-induced colitis in rats.

### 2.10. AEE Improved Inflammation and Intestinal Barrier Damage in LPS-Induced Rats

**Figure 11:**
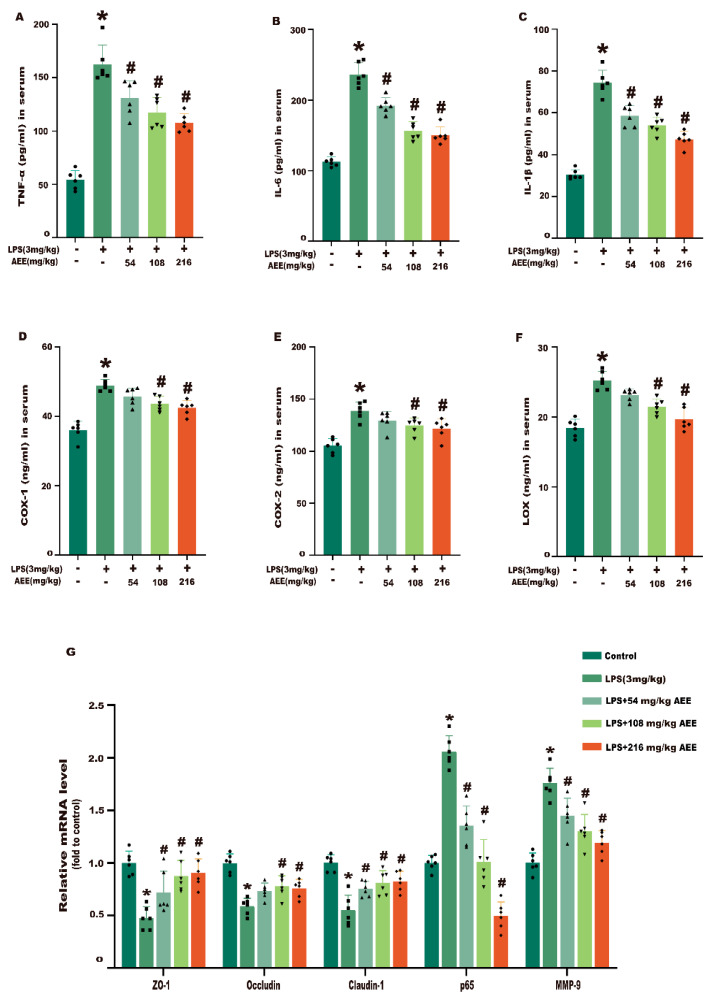
Effects of AEE on the inhibition of LPS-induced colonic inflammation and breakdown of TJs in rats. (**A**) TNF-α, (**B**) IL-6, (**C**) IL-1β, (**D**) COX-1, (**E**) COX-2, and (**F**) LOX. (**G**) Relative mRNA expression of ZO-1, Occludin, Claudin-1, NF-κB, and MMP-9 in colonic tissues. Values are presented as the means ± SD where applicable (*n* = 6). * *p* < 0.05 compared to control group, ^#^ *p* < 0.05 compared to LPS group. One-way ANOVA followed by Duncan’s multiple comparisons.

In this experiment, ELISA was used to detect inflammatory factors in serum to assess the inhibitory effect of AEE on inflammation in LPS-induced colitis rats. As shown in Figure 11A–F, compared with the control group, the levels of TNF-α, IL-6, IL-1β, COX-1, COX-2, and LOX were significantly higher in the LPS group (*p* < 0.05). The levels of TNF-α, IL-6, IL-1β, COX-1, COX-2, and LOX were significantly lower in the AEE group compared with the LPS group (*p* < 0.05), which showed a certain dose–effect relationship. Next, RT-qPCR was performed to detect the intestinal barrier-related genes to assess the effect of AEE on the improvement in the intestinal barrier integrity in LPS-induced colitis rats. As shown in Figure 11G, the mRNA levels of ZO-1, Occludin, Claudin-1, p65, and MMP-9 were significantly higher in the colonic tissues of LPS-treated rats than in the control group (*p* < 0.05). Compared with the LPS group, the mRNA levels of ZO-1, Occludin, Claudin-1, p65, and MMP-9 were significantly lower in the colonic tissues of rats in the AEE group (*p* < 0.05). These results indicate that AEE reduced the levels of inflammatory factors and improved intestinal barrier damage in LPS-induced colitis rats.

### 2.11. AEE Improved the Gut Microbiota Structure in LPS-Treated Rats

#### 2.11.1. Phylum Level

**Figure 12:**
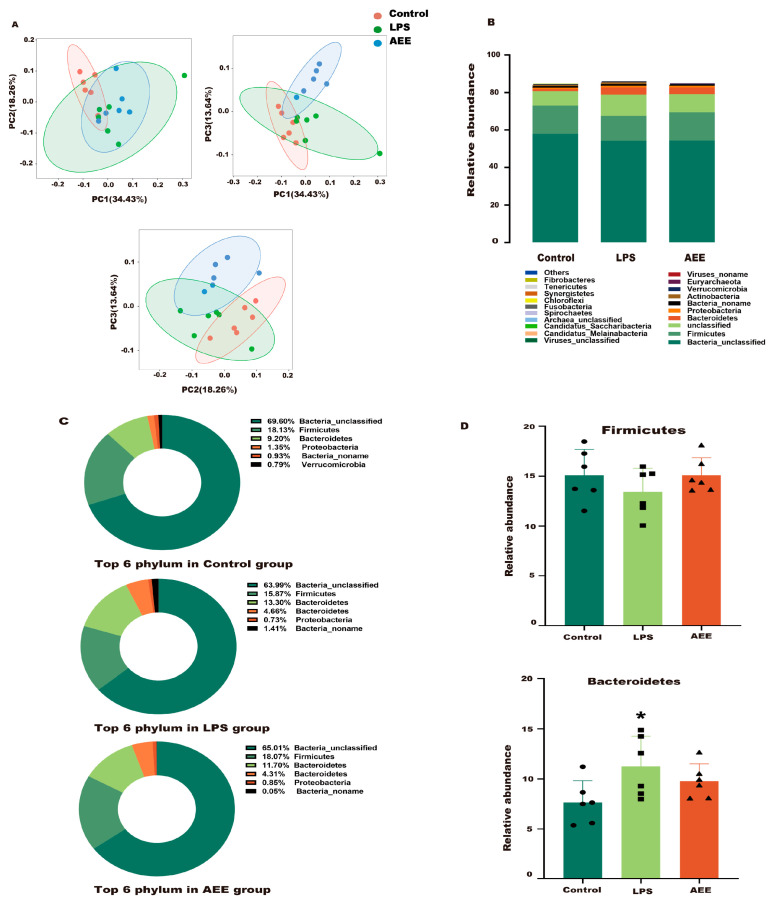
Taxonomic profile of the cecum microbiota at the phylum level. (**A**) PCA of microbial communities at the phylum level. (**B**) Relative abundance of the cecum microbiome at the phylum level. (**C**) Proportion of the top 6 phyla in the control, LPS, and AEE groups. (**D**) Relative abundance of Firmicutes and Bacteroidetes in the control, LPS, and AEE groups. Values are presented as the means ± SD where applicable (*n* = 6). * *p* < 0.05 compared to control group. Student’s *t*-test.

To assess the effects of AEE on the microbial community of LPS-treated rats, this study compared the microbial taxa of rats in three experiment groups at the phylum level. As shown in Figure 12A, PCA showed a clear demarcation between the three groups (control, LPS, and AEE groups), which indicated a significant change in the microbial community. As shown in Figure 12B,C, *Bacteroidetes* and *Firmicutes* were the superior phyla, accounting for more than 90% of the total microbial community. Compared with the control group, *Firmicutes* was reduced in the LPS group (*p* > 0.05) and the abundance of the *Bacteroidetes* was increased (*p* < 0.05). Compared with the LPS group, the AEE group showed an increasing trend in the content of the *Firmicutes* (*p* > 0.05) and a decreasing trend in the abundance of the *Bacteroidetes* (*p* > 0.05) (Figure 12D).

#### 2.11.2. Genus Level

**Figure 13:**
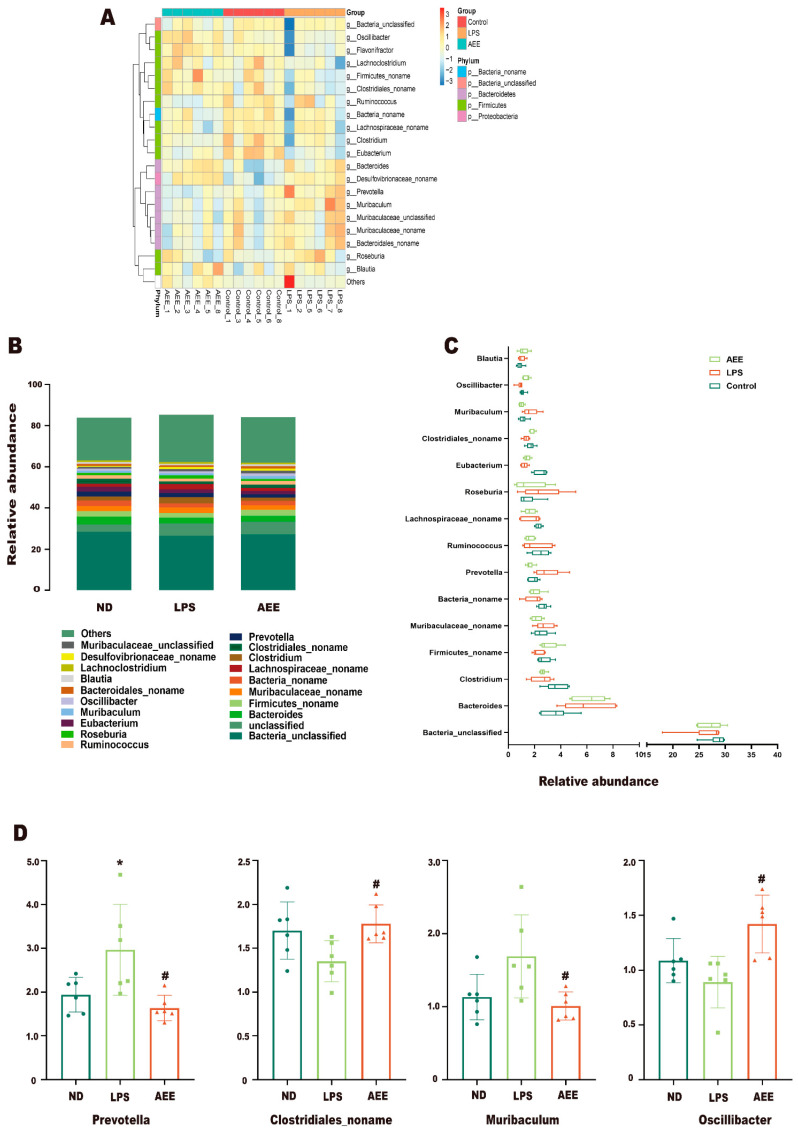
Taxonomic profile of the cecum microbiota at the genus level. (**A**) Heatmap for relative abundance of cecum microbiome at the genus level in the three groups (control, LPS, and AEE groups). (**B**) Relative abundance of the cecum microbiome at the genus level. (**C**) Comparisons of the relative abundance at the genus level in the three groups (control, LPS, and AEE groups). (**D**) Relative abundance of *Prevotella*, *Clostridiales_noname*, *Muribaculum* and *Oscillibacter* at genus level. Values are presented as the means ± SD where applicable (*n* = 6). * *p* < 0.05 compared to control group, ^#^ *p* < 0.05 compared to LPS group. Student’s *t*-test.

As shown in Figure 13A, the microbial taxa at the genus level changed in the three experimental groups. As shown in Figure 13B, these groups mainly included *Bacteria_unclassified*, *unclassified*, *Firmicutes_noname*, *Muribaculaceae_noname*, *Bacteria_noname*, *Lachnospiraceae_noname*, *Clostridiales_noname*, *Prevotella*, *Ruminococcus*, and *Roseburia*. These were the dominant flora in the ND, HFD, and AEE groups. As shown in Figure 13C,D, compared with the control group, the relative abundances of *Clostridium*, *Firmicutes_noname*, *Bacteria_noname*, *Eubacterium*, *Clostridiales_noname*, and *Oscillibacter* decreased in the LPS group, while the relative abundance of *Bacteroides*, *Muribaculaceae_noname*, *Prevotella*, *Roseburia*, and *Muribaculum* increased. In contrast, AEE treatment could restore most of them to normal levels.

#### 2.11.3. Species Level

**Figure 14:**
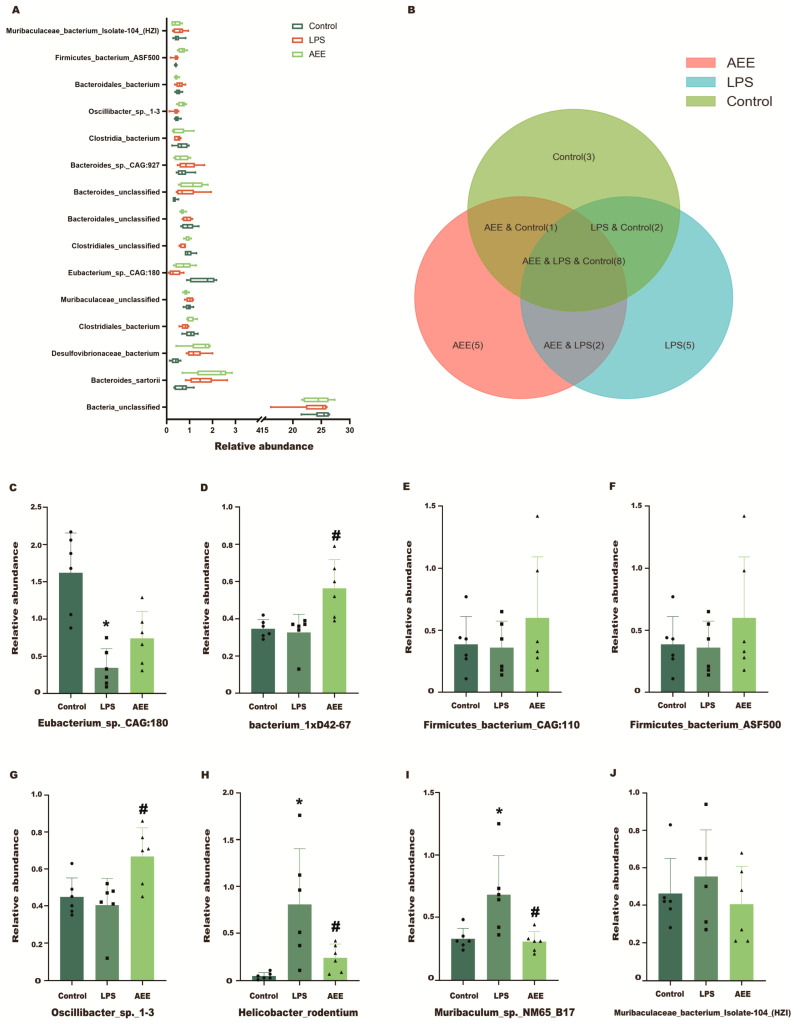
Taxonomic profile of the cecum microbiota at the species level. (**A**) Comparisons of the relative abundance at the species level in the three groups (control, LPS and AEE groups). (**B**) UpSet plot for relative abundance of cecum microbiome at the species level in the three groups (control, LPS, and AEE groups). (**C**–**J**) Relative abundance of bacteria in the control, LPS, and AEE groups. Values are presented as the means ± SD where applicable (*n* = 6). * *p* < 0.05 compared to control group, ^#^ *p* < 0.05 compared to LPS group. Student’s *t*-test.

Next, this experiment wanted to investigate the changes at the species level of bacteria in the three experimental groups. Figure 14A shows the changes in the relative abundance of the first 15 bacterial species in the three experimental groups. Among them, the relative abundance changed significantly in *Bacteroides_sartorii*, *Desulfovibrionaceae_bacterium*, Clostridiales_bacterium, *Eubacterium_sp._CAG:180*, *Clostridiales_unclassified*, *Oscillibacter_sp._1-3*, and *Firmicutes_bacterium_ASF500*. Next, the UpSet plot method was performed to select bacteria in the obtained bacterial species-level data table. The results shown in Figure 14B show that there were eight bacterial classes in total in the three experimental groups, one in the control and AEE groups, two in the control and LPS groups, and two in the AEE and LPS groups. As shown in Figure 14C–J, compared with the control group, the relative abundance of *Eubacterium_sp._CAG:180* decreased and the relative abundance of *Helicobacter_rodentium*, *Muribaculum_sp._NM65_B17*, and *Muribaculaceae_bacterium_Isolate-104_(HZI)* increased in the LPS group. Compared with the LPS group, the relative abundance of *Helicobacter_rodentium*, *Muribaculum_sp._NM65_B17*, and *Muribaculaceae_bacterium_Isolate-104_(HZI)* decreased and the relative abundance of *Eubacterium_sp._CAG:180*, *bacterium_1xD42-67*, *Firmicutes_bacterium_CAG:110*, and *Oscillibacter_sp._1-3* increased in the AEE group.

#### 2.11.4. Differential Species Analysis

In this study, linear discriminant analysis (LDA) was performed to identify the bacterial classes with the most significant differences in abundance, as shown in Figure S6. The control group had c_*Bacteria_unclassified*, p_*Bacteria_unclassified*, f__*unclassified*, o__*unclassified*, s__*unclassified*; the LPS group had p_*Proteobacteria*, d_*Bacteria*, c_*Gammaproteobacteria*, f_*Prevotellaceae*, and g_*Prevotella*; and the AEE group had g_*Bacteroides*, f_*Bacteroidaceae*, s_*Bacteroides_sartorii*, c_*Deltaproteobacteria*, and o_*Desulfovibrionales*. The changes in microbial classes were shown at the phylum level, and Figure S7A,B show the changes in p_*Firmicutes*, p_*Proteobacteria*, p_*Bacteroidetes*, p_*Actinobacteria*, p_*Viruses_noname*, p_*Bacteria_noname*, p_*Euryarchaeota*, p_*Cyanobacteria*, p_*Spirochaetes*, and p_*Planctomycetes*. These results indicate that AEE could partly reverse the dysbiosis of gut flora in LPS-treated rats.

#### 2.11.5. Functional Level Analysis of Cecum Microbes by Metagenome

As shown in Figure S8A,B, the control and LPS groups identified a total of 805,278 differentially expressed genes (DEGs). A total of 238,279 upregulated genes and 566,999 downregulated genes were identified when comparing the control group with the LPS group. A total of 393,569 DEGs with 262,696 upregulated genes and 130,873 downregulated genes were identified between the AEE group and LPS group. The Venn diagram of the DEGs showed that there were 2,784,285 common DEGs to the three groups. GO enrichment analysis provides all GO terms that are significantly enriched in DEGs compared to the genomic background and selects DEGs that correspond to biological functions. Pathway enrichment analysis can aggregate the different signaling pathways involved in DEGs. KEGG is the main public database for path analysis. Next, this study used two functional annotation systems to analyze the function of cecum microbes (GO and KEGG). As shown in Figure S8C,D, GO enrichment analysis DEGs were mainly enriched in the arginine biosynthetic process, biological process, plasma membrane, phosphorelay sensor kinase activity, and oxidoreductase activity. KEGG enrichment analysis DEGs were mainly enriched in pentose and glucuronate interconversions; quorum sensing; cysteine and methionine metabolism; pentose phosphate pathway; 2-oxocarboxylic acid metabolism; glycerolipid metabolism; butanoate metabolism; and ABC transporters. As shown in Figure S9A–C, the results of the KEGG analysis at the metagenome level showed that genes were mainly enriched in lipid metabolism; metabolism of cofactors and vitamins; glycan biosynthesis and metabolism; energy metabolism; carbohydrate metabolism; global and overview maps; amino acid metabolism; replication and repair; membrane transport; and signal transduction. The KEGG pathway analysis showed that the metabolism pathway was the most significant, with most genes enriched in global and overview maps, translation, membrane transport, energy metabolism, and the metabolism of cofactors and vitamins. As shown in Figure S9D,E, CAZy enrichment analysis DEGs were mainly enriched in glycoside hydrolases, glycosy transferases, carbohydrate-binding modules, and carbohydrate esterases. These indicated that AEE improved the structure of LPS-induced cecum microbes in rats, which might be related to the regulation of glucose metabolism and energy metabolism of cecum microbes.

## 3. Discussion

The intestinal mucosal barrier effectively prevents the entry of pathogens and actively participates in the innate and acquired immune response through the secretion of multiple inflammatory mediators [22]. Inflammatory responses’ occurrence and development are closely associated with oxidative stress. Oxidative stress is an imbalance of oxidative and antioxidant systems that leads to oxidative damage in the body [23]. Previous studies have shown that AEE can improve oxidative stress, mitochondrial dysfunction, and immune–inflammatory responses [15,19,24,25]. Several studies have demonstrated that Caco-2 cells are suitable for studies related to intestinal inflammation [8,26]. Therefore, this experiment evaluated the inhibitory effect of AEE on the intestinal inflammatory response with the LPS-induced Caco-2 cell inflammation model and rat intestinal inflammation model.

Firstly, this experiment used a model of LPS-induced inflammation in Caco-2 cells. In normal physiological conditions, the level of ROS in the body is in a state of equilibrium. When the body is in a state of oxidative stress, the level of ROS will increase and thereby damage the health of the body [27]. Pro-inflammatory cytokines play an essential role in the regulation of intestinal immunity, and among them, IL-1β, TNF-α, and IL-6 abnormalities are considered to be important in the pathogenesis of inflammatory bowel disease [28]. COX can metabolize arachidonic acid (AA) to prostaglandins and thereby participate in the inflammatory response, mainly in two isomeric forms. COX-1 is mainly involved in prostaglandin synthesis, which plays an essential role in the maintenance of body homeostasis. COX-2 is an inducible enzyme that can be induced by pro-inflammatory cytokines, which are mainly expressed at sites of tissue damage and inflammation [29,30]. Lipoxygenase (LOX) also metabolizes AA to leukotrienes (LTs) [31], which are considered to be a key mediator of allergic and inflammatory diseases [32]. In previous studies, chitosan nanoparticles (CNPs) [33], paeoniflorin (PF) [8], and luteolin [34] could inhibit the LPS-induced inflammatory response by decreasing the secretion of pro-inflammatory cytokines and ROS in Caco-2 cells. Consistent with these studies, this study found that the levels of ROS, TNF-α, IL-6, IL-1β, COX-1, COX-2, and LOX were significantly increased in Caco-2 cells after LPS inducement, and AEE could inhibit this phenomenon. This indicates that AEE could alleviate LPS-induced oxidative stress and inflammatory responses in Caco-2 cells.

The intestinal mucosal barrier function is essential for nutrient absorption and maintenance of intestinal homeostasis, which consists mainly of TJ proteins including ZO-1, Occludin, and Claudin. The expression reduction in these TJ proteins is a major factor contributing to their effect on intestinal barrier function [35,36]. Several studies have shown that inflammatory cytokines lead to damaged TJs and impaired intestinal barriers [37,38,39]. Aspirin protects the expression of endothelial junction proteins, including ZO-1 and ZO-2, by inhibiting the release of ROS, thereby restoring tight junction proteins and permeability [40]. Eugenol could attenuate the LPS-induced inflammatory response in IPEC-J2 cells and enhance intestinal barrier function [41]. AEE is a novel medicinal compound synthesized by esterification of aspirin with eugenol using the twin drug principle. Therefore, it is speculated that AEE may also have some preventive and palliative value in intestinal inflammation. Firstly, the study observed the morphology of Caco-2 cells using an inverted microscope. The results showed that the Caco-2 cytoskeleton was disrupted after LPS inducement, while AEE pretreatment could effectively alleviate this destruction. TEER and LY-flux are methods to detect cellular TJs and are commonly used to assess the integrity of cellular TJs [42,43]. Previous studies have found that tryptophan, glutamate, and collagen peptides could regulate changes in TEER and LY-flux and had a role in ameliorating intestinal barrier damage [44,45,46]. Dietary l-Trp could attenuate the LPS-induced decrease in TEER and increase in LY-flux in Caco-2 cell monolayers, thereby protecting the integrity of the intestinal barrier [47]. Consistent with these studies, the present study found a decrease in TEER and an increase in LY-flux in Caco-2 cell monolayers after LPS treatment, while AEE pretreatment attenuated this phenomenon. Intestinal epithelial monolayer permeability and integrity are related to intestinal TJ proteins, and the destruction of intestinal TJs will lead to increased intestinal permeability and consequently to intestinal barrier dysfunction [39,48]. Therefore, this experiment examined the changes in the expression of TJ proteins after AEE pretreatment. Previous studies have shown that *Glycyrrhiza* polysaccharide promotes the expression of intestinal TJ proteins (Occludin, Claudin-1, and ZO-1) and maintains intestinal epithelial TJs [49]. Luteolin could inhibit the reduction in ZO-1, Occludin, and Claudin-1 protein expression in BDE-209-stimulated Caco-2 cells, thereby protecting the integrity of the intestinal barrier [34]. Similar to these studies, this study found that the expression of TJ proteins (ZO-1, Occludin, and Claudin-1) was reduced in Caco-2 cells after LPS treatment, and AEE pretreatment could inhibit the LPS-induced decrease in TJ protein expression in Caco-2 cells to some extent.

NF-κB is a transcription factor with a key role in inflammation and is involved in the response to foreign cellular irritants [50,51]. NF-κB is also thought to be a major factor involved in TJ protein expression and intestinal epithelial permeability and plays an essential role in the development of inflammatory bowel disease by regulating inflammatory factors [52,53,54]. MMPs are zinc-dependent neutral endopeptidases involved in a variety of diseases including inflammation and cancer, with the main pathology leading to extracellular degradation of matrix proteins. MMPs mediate inflammation mainly by MMP-9 [55]. In previous studies, *Lycium barbarum* polysaccharides significantly inhibited the TNF-α-induced activation of p-IκBα and NF-κBp65, thereby suppressing the release of inflammatory cytokines [56]. Phycocyanobilin could exert anti-inflammatory effects by inhibiting the activation of NF-κB [57]. Paeoniflorin significantly inhibited LPS-induced NF-κB activation and the expression of MMP-9 in Caco-2 cells [8]. Consistent with these studies, AEE could significantly inhibit LPS-induced NF-κB activation and MMP-9 expression in Caco-2 cells. Also, studies have shown that intestinal inflammation leads to elevated MMP-9 expression, while elevated MMP-9 was not the cause of intestinal inflammation [58,59]. MMP-9 is produced as an inactive zymogen (proMMP-9), and cleavage of the prepeptide structural domain yields active MMP-9 [60]. Neutrophil gelatinase-associated lipocalin (NGAL) is a 25 kDa-sized small-molecule protein, belonging to the lipocalin family, which is synthesized and secreted by intestinal epithelial cells, endothelial cells, and neutrophils. NGAL could bind to the inactive precursor MMP-9 (proMMP-9) through disulfide bonds to form a dimer, thereby reducing MMP-9 degradation and promoting MMP-9 activation [61]. Therefore, the relationship between AEE and MMP-9 and proMMP-9 needs to be explored in depth subsequently.

To further investigate the inhibitory effect of AEE on intestinal inflammation, this experiment used the LPS-induced Wistar rats to establish an in vivo intestinal inflammation model. The results showed that AEE improved weight loss, diarrhea, reduced intestinal muscle thickness, and intestinal villi damage in LPS-induced rats. In addition, AEE also improved spleen hypertrophy in rats with intestinal inflammation caused by LPS. In previous studies, 5-ASA could improve DSS-induced colitis in mice and also reduce the spleen index [21]. *Glycyrrhiza* polysaccharide could improve LPS-induced colonic inflammation in mice and reduce the spleen index [49]. This experiment’s results were similar. In previous studies, *Herba Origani* significantly reduced the levels of IL-1β and TNF-α in a dose-dependent manner and upregulated the protein expression of Occludin and Claudin1 in colonic tissues of DSS-induced mice [62]. Polysaccharides could inhibit the decrease in ZO-1 and Occludin gene expression in LPS-stimulated Caco-2 cells, thereby maintaining the intestinal epithelial integrity in DSS-induced colitis mice [63]. Similar to these findings, LPS treatment significantly increased the levels of TNF-α, IL-6, IL-1β, COX-1, COX-2, and LOX in rat serum; decreased the relative mRNA expression of ZO-1, Occludin, and Claudin-1; and increased the relative mRNA expression of NF-κB and MMP-9 in rat colon tissue. AEE pretreatment could inhibit this phenomenon to some extent. The above results were consistent with the results of in vitro Caco-2 cell experiments, which indicated that AEE could alleviate the intestinal barrier damage caused by intestinal inflammation.

Intestinal inflammation is closely related to dysbiosis of the gut flora [64]. Studies have confirmed large differences in the composition of intestinal microbes between patients with enteritis and healthy individuals [65]. The *Firmicutes* and *Bacteroidetes* were the major communities in the intestine, accounting for 90% of the intestinal microbiota [66]. *Gastrodia elata* polysaccharides could reverse DSS leading to the abundance of *Firmicutes* decreasing and the abundance of *Bacteroidetes* increasing in mice [67]. Consistent with these results, the results in this study showed that the structure of gut flora in rats changed after LPS treatment, with the abundance of *Firmicutes* decreasing and the abundance of *Bacteroidetes* increasing. AEE inhibited the LPS-induced changes in rat gut flora, which indicated that AEE regulated the LPS-induced dysbiosis in rat gut flora. Intestinal inflammation could lead to changes in the community structure of *Firmicutes* and *Bacteroidetes*, thus affecting the absorption of bile acids (BAs) and further aggravating intestinal inflammation [68]. BA receptors include both farnesoid X receptor (FXR) and Takeda G protein-coupled receptor 5 (TGR5), which can affect the body’s BA metabolism and systemic inflammatory response [69]. Liver TGR5 activation could trigger inflammatory responses by p-ERK signaling [70]. Research has found that 5-ASA reduced levels of liver TGR5 and p-ERK proteins and helped to alleviate DSS-induced systemic inflammation in mice [21]. Similar to these results, AEE induced the expression of BA synthase cholesterol 7α-hydroxylase (CYP7A1) by inhibiting BA nuclear receptor FXR in the liver [71].

*Clostridiales* could induce the upregulation of T cells and stimulate the production of anti-inflammatory cytokines [72]. Peanut skin procyanidins could increase the abundance of *Oscillibacter* in DSS-treated mice, thereby improving intestinal inflammation [73]. *Prevotella* could alter the homeostasis of the gut flora, leading to a decrease in short-chain fatty acids, which can exacerbate intestinal inflammation [74]. Similar to these findings, AEE increased the abundance of *Clostridiales* and *Oscillibacter* and reduced the abundance of *Prevotella*. Fucoidan could increase the abundance of *Muribaculum* in DSS-treated mice, thereby improving intestinal inflammation [75]. This result was in contrast with the results of AEE on *Muribaculum*. The functional metagenome analysis showed that the metabolism pathway was the most significant, which included energy metabolism, metabolism of cofactors and vitamins, glycoside hydrolases, glycosy transferases, carbohydrate-binding modules, and carbohydrate esterases. These indicated that AEE improved the structure of LPS-induced cecum microbes in rats, which might be related to the regulation of glucose metabolism and energy metabolism of cecum microbes.

In conclusion, AEE may exert a protective effect against intestinal inflammation induced by LPS, thus helping to mitigate damage to the intestinal barrier. AEE could have positive effects on intestinal inflammation-related diseases. In subsequent studies, we will further explore the specific effects of AEE on the gut flora of rats with enteritis, further validate its function in vivo, and explore the relationship between AEE and MMP-9 in more depth.

## 4. Materials and Methods

### 4.1. Materials

Caco-2 cells were obtained from the American Type Culture Collection (ATCC, Manassas, VA, USA). AEE with a purity of 99.5% was prepared at the Lanzhou Institute of Husbandry and Pharmaceutical Sciences of CAAS, Lanzhou, China. LPS from *Escherichia coli* 055: B5 was obtained from Solarbio (Beijing, China). Trypsin-EDTA (0.05%), modified eagle medium (MEM), Hanks buffer (HBSS), non-essential amino acids (NEAAs), glutamine, sodium pyruvate 100 mM solution, and fetal bovine serum were from Gibco (Grand Island, NY, USA). ELISA kits were produced by Shanghai Mlbio for the detection of GSH (mlsh0038), SOD (mlsh0053), LDH (mlsh1050), IL-6 (ml058097-1), IL-1β (ml058059-1), TNF-α (mlC30498-1), COX-1 (ml038215), COX-2 (ml062904), and LOX (ml060424) in cells (Shanghai, China). ELISA kits were produced by Shanghai Mlbio for the detection of IL-6 (ml102828), IL-1β (ml037361), TNF-α (ml002859), COX-1 (ml0323908), COX-2 (ml058808-C), and LOX (ml556645-C) in rats (Shanghai, China). Cell counting kit-8 (C0038), DAPI (C1002), and the Reactive Oxygen Species Assay Kit (S0033M) were purchased from Beyotime (Shanghai, China). RIPA (78,501) and inhibitor (1,861,280) were supplied by Thermo Fisher Scientific (Thermo Fisher Scientific, Waltham, MA, USA). PVDF membrane (IPVH00010) was purchased from Merck (Shanghai, China). The BCA protein assay kit (PC0020), phosphate-buffered saline (PBS, P1020), precast protein gel (PG42010-N), protein marker (PR1910, PR1920), and lucifer yellow (LY, IL2610) were supplied by Solarbio (Beijing, China). The Transwell-TM cell culture plate (0.4 μm, Model 3460) and cell culture plate were purchased from Corning (Beijing, China). ZO-1 antibody (ab276131), Occludin antibody (ab216327), Claudin-1 antibody (ab15098), MMP-9 antibody (ab76003), NF-κB p65 antibody (ab32536), NF-κB p-p65 antibody (ab76302), Alexa Fluor^®^ 488 (ab150077), and β-actin antibody (ab8227) were from Abcam (Cambridge, MA, USA). Carboxymethylcellulose sodium (CMC-Na) was supplied by Tianjin Chemical Reagent Company (Tianjin, China). The other analytical-grade reagents were purchased from the Sinopharm group (Shanghai, China).

### 4.2. Cell Culture and Treatments

Caco-2 cells were cultured as described previously [22]. Briefly, Caco-2 cells (1 × 10^5^ cells/mL) were cultured in 20% FBS, 1% glutamine, 1% sodium pyruvate, 1% NEAA, and 77% MEM media supplemented at 37 °C under humidified atmospheric conditions containing 5% CO_2_. Caco-2 cells were trypsinized and passaged at a ratio of 1:3. Then, the groups were randomly divided into three groups, namely, the control, LPS, and AEE pretreatment groups.

### 4.3. Cell Viability

Before the start of this experiment, the effect of the drug on the viability of the Caco-2 cells was detected to screen the appropriate drug concentration for subsequent experiments. Cell viability was examined using a CCK-8 cell viability assay kit (Beyotime, Shanghai, China), according to the manufacturer’s instructions.

### 4.4. AEE Treatment and LPS Stimulation

Firstly, Caco-2 cell monolayers were established as described previously [22]. Briefly, Caco-2 cells (1 × 10^5^ cells/mL) were cultured in 12-well Transwell-TM cell culture plates, and the culture medium was replaced after 24 h, every other day for the first week, and then every day thereafter. Caco-2 cells were cultured continuously under standard cell culture conditions for 22 days, and the cell state was homogeneous and dense, with the formation of tightly connected monolayers of cells. The fully differentiated Caco-2 cell monolayer cell model was treated with AEE for 24 h, followed by stimulation with LPS for 24 h: MEM (blank), LPS (100 μg·mL^−1^), AEE (8 μM) + LPS (100 μg·mL^−1^), AEE (32 μM) + LPS (100 μg·mL^−1^), and AEE (64 μM) + LPS (100 μg·mL^−1^) combinations.

### 4.5. Measurement of Trans-Epithelial Monolayer Resistance

The trans-epithelial monolayer resistance (TEER) measurement of Caco-2 cell monolayer models was conducted using a cell resistance meter (MERS00002, Merck, Rahway, NJ, USA). Each well was measured 3 times and calculated as an average. The experiments only use monolayers of cells with a resistance greater than 500 Ω·cm^2^.

TEER was calculated as follows:

$$\mathrm{TEER}(\Omega \cdot {\mathrm{cm}}^{2})=\left[\mathrm{TEER}(\Omega )-{\mathrm{TEER}}_{\mathrm{background}}\left(\Omega \right)\right]\times A({\mathrm{cm}}^{2})$$

where A is the cell monolayer membrane area (1.12 cm^2^).

### 4.6. Measurement of Paracellular Permeability

Lucifer yellow (LY) solution was often used to test the integrity of the Caco-2 cell monolayer model due to its poor transport and uptake in the Caco-2 cell monolayer model. An amount of 0.5 mL of 100 μg·mL^−1^ LY solution was added to the AP side as the donor side, while 1.5 mL HBSS was added to the BL side as the recipient side. After incubation in the cell incubator for 2 h, 100 μL of the solution from the BL side was taken and measured at excitation/emission wavelengths of 410/520 nm.

### 4.7. Measurement of LDH, ROS, SOD, and GSH in Cell Supernatant

The levels of LDH (lactate dehydrogenase), ROS (reactive oxygen species), SOD (superoxide dismutase), and GSH (glutathione) in the cell supernatant were measured using the appropriate kits according to the manufacturer’s instructions.

### 4.8. Protein Extraction and Western Blotting

Caco-2 cells were treated with a medium containing different concentrations of AEE for 24 h and then treated with LPS for 24 h. Total protein was extracted using RIPA buffer containing a protease inhibitor, and cell lysates were centrifuged at 14,000× *g* for 10 min at 4 °C to remove cell debris. The supernatant was collected and the protein concentration was measured according to the BCA protein assay kit. The protein samples in equal amounts (20 μg) were electrophoresed in 10% SDS-PAGE gel and then transferred to PVDF. The PVDF was blocked in the blocking solution for 20 min at ambient temperature and then incubated with the primary antibody overnight at 4 °C. The following antibody dilutions were used: ZO-1 (1:1000), Occludin (1:1000), Claudin-1 (1:500), MMP-9 (1:5000), NF-κB p65 (1:5000), NF-κB p-p65 (1:1000), and β-actin (1:5000). Then, the samples were incubated with the secondary antibody for 2 h at ambient temperature. Protein bands were visualized using an enhanced chemiluminescence kit and detected using the BIO-RAD imaging system (BIO-RAD, Hercules, CA, USA). Signal plots were processed using Image J 1.8.0 software. The target protein expression was normalized using β-actin and expressed as the mean fold change relative to the control group.

### 4.9. Immunofluorescence

Caco-2 cells were first inoculated in cell culture plates containing a round coverslip and cultured for a period of time. After a series of treatments, briefly, Caco-2 cells were pretreated with AEE for 24 h and then stimulated with LPS for 24 h.

The Caco-2 cells were subject to immunofluorescence. Caco-2 cells were first washed twice with PBS. After fixation, permeabilization, and closure, the cells were incubated overnight at 4 °C with primary antibodies and exposed to FITC-coupled secondary antibodies for 1 h. The following antibody dilutions were used: ZO-1 (1:200), Occludin (1:100), Claudin-1 (1:200), MMP-9 (1:500), NF-κB p65 (1:250), and Alexa Fluor^®^ 488 (1:1000). Caco-2 cells were further stained with DAPI for 5 min, and the staining of the cells was observed under a Zeiss confocal laser scanning microscope (LSM 800, Zeiss, Germany). Image analysis was performed using Image J (NIH Image J system, Bethesda, MD, USA).

### 4.10. Metabolomics Analysis

#### 4.10.1. Cell Collection and Processing

After collecting the treated cell culture supernatant, the cells were quickly blunted by adding 1 mL of ice-methanol and then placed in a −80 °C freezer for 10 min. The cells were scraped off with a cell scraper, repeatedly freeze–thawed (liquid nitrogen quick freeze, 37 °C quick thaw) twice to break up the cells, centrifuged at 14,000× *g* for 10 min at 4 °C, and then the supernatant was collected. Quality control (QC) samples were prepared by aspirating 20 μL from each sample and mixing them. The protein was precipitated by adding ice-methanol (3:1, *v*/*v*) to the sample and QC samples followed by centrifugation at 14,000× *g* for 10 min at 4 °C. The supernatant was filtered through the 0.22 μm filter membrane and subsequently analyzed by ultra-high-performance liquid chromatography combined with quadrupole time-of-flight tandem mass spectrometry (UPLC-Q-TOF-MS/MS, 6530, Agilent Technologies, Santa Clara, CA, USA).

#### 4.10.2. UPLC-QTOF-MS/MS Conditions

The UPLC/QTOF-MS coupling technique was used for the detection of small-molecule metabolites in cell samples. The chromatography system was set up with the following parameters: The column was an Agilent ZORBAX SB-C18 (2.1 × 150 mm, 1.8 μm) and the column temperature was 35 °C. The injection volume was 2 μL and the autosampler temperature was set at 4 °C. Mobile phase: 0.1% formic acid in water (*v*/*v*) for A and 0.1% formic acid in acetonitrile (V/V) for B at a flow rate of 0.25 mL·min^−1^. Gradient elution procedures are shown in Table S3. Mass spectrometry was performed with electrospray ionization in both positive (ESI^+^) and negative (ESI^−^) ion modes. The mass spectrometer system was set up with the following parameters: scan range 40–1000 *m*/*z*; acquisition rate 1 spectra/s; nitrogen (dry gas) temperature 350 °C; flow rate 10 L·min^−1^; nebulizer pressure 45 psi; capillary voltage of 4000 V for ESI^+^ and 3500 KV for ESI^−^; and fragmentation voltage 135 V.

#### 4.10.3. Metabolomics Data Analysis

Metabolite quantification was performed by using the ABF Converter software 4.0.0 to convert raw data into “Analysis Base File” (ABF) files. For data processing, the MSDIAL 2.2.62 software was used for peak detection, deconvolution, and alignment. The obtained data set was analyzed by SIMCA software (version 14.0, Umetrics AB, Sweden) for multivariate statistics, including unsupervised and supervised learning methods. The results were presented as principal component analysis (PCA) score plots and partial least squares discriminant analysis (OPLS-DA) score plots, respectively. The OPLS-DA model was also evaluated using the R^2^X, R^2^Y, and Q^2^ parameters. Finally, the metabolites with VIP > 1 and *p* < 0.05 were screened for metabolite variability analysis in different groups. Based on the above conditions, differential metabolites were screened and analyzed by secondary targeted mass spectrometry using a UPLC/QTOF-MS analysis system to obtain information about their precise fragment ions. The information on the mother ion and fragment ions was used to search the spectral libraries in the Human Metabolome Database (HMDB), Metlin database, and Massbank to identify the differential metabolites. The information on the differential metabolites was used to analyze the relevant metabolic pathways using MetaboAnalyst online software (http://www.metaboanalyst.ca) and the Kyoto Encyclopedia of Genes and Genomes (KEGG) database (http://www.genome.jp/kegg/mapper.html).

### 4.11. Drug Preparation in the Animal Study

The first step was to prepare a 0.5% CMC-Na solution. AEE was dissolved in 0.5% CMC-Na solution to form the storage suspension solution of 108 mg·mL^−1^. LPS was dissolved in saline to form the storage solution of 3 mg·mL^−1^. Based on the recorded body weight of the rats, the dosage of the drug administered was calculated for AEE (54, 108, and 216 mg·kg^−1^ body weight) and LPS (3 mg·kg^−1^ body weight).

### 4.12. Animal Experiment

Forty male specific pathogen-free Wistar rats (7 weeks old) weighing 160~180 g were provided by the Lanzhou Veterinary Research Institute, Chinese Academy of Agricultural Sciences (Lanzhou, China), and housed in an SPF-rated laboratory under controlled relative humidity (55–65%), a 12 h light/dark cycle, and a temperature of 24 ± 2 °C, and food and water were offered ad libitum. All experimental protocols and procedures were approved by the Institutional Animal Care and Use Committee of the Lanzhou Institute of Husbandry and Pharmaceutical Science of the Chinese Academy of Agricultural Sciences (Approval No. 2021-011). Animal welfare and experimental procedures were performed strictly according to the Guidelines for the Care and Use of Laboratory Animals issued by the US National Institutes of Health. The rats were randomly divided into five groups (*n* = 8), namely, the control, model (LPS group), AEE + LPS low (AEE, 54 mg·kg^−1^), AEE + LPS middle (AEE, 108 mg·kg^−1^ ), and AEE + LPS high group (AEE, 216 mg·kg^−1^). The Wistar rat intestinal injury model was established by the intraperitoneal injection (*i.p.*) of LPS (3 mg·kg^−1^). Briefly, Wistar rats were given either the vehicle alone (0.5% Carboxymethylcellulose sodium, CMC-Na) or the vehicle combination containing AEE (54, 108, and 216 mg·kg^−1^·d^−1^) for 14 d. Then, all groups of rats, except those in the normal group, were injected with LPS continuously for 3 days. On the 18th day of the experiment, after all rats had fasted for 12 h, the animals were anesthetized with 80 mg·kg^−1^ sodium pentobarbital by *i.p*. The blood samples were collected for further analysis through a puncture of the heart. Then, colonic intestinal tissue and cecum contents were carefully obtained, snap-frozen in liquid nitrogen, and stored at −80 °C.

### 4.13. Measurement of Disease Activity Index (DAI) Scores and Immune Organ Index

During the animal experiments, the body weight, anal bleeding, and fecal consistency of the rats were recorded daily. DAI scores were assessed according to the rules in Table S1. Body weight was recorded before execution. After execution, spleen tissue was collected and weighed. The spleen tissues were collected to calculate the immune organ index. Immune organ index = spleen weight (mg)/rat body weight (g).

### 4.14. Measurement of Inflammatory Factors in Serum and Cell Supernatant

The levels of IL-6 (Interleukin-6), IL-1β (Interleukin-1β), TNF-α (Tumor necrosis factor-α), COX-1 (Cyclooxygenase-1), COX-2 (Cyclooxygenase-2), and LOX (Lipoxygenase) in the serum and the cell supernatant were measured using the appropriate kits according to the manufacturer’s instructions.

### 4.15. Histopathological Analysis

Fresh colonic tissue samples were fixed in 10% formalin buffer solution, dehydrated in ethanol, embedded in paraffin, and cut into 5 μm slices. Then, they were stained using standard hematoxylin–eosin (HE), followed by pathological observation. Histological analysis was performed according to standard procedures. The HE trial was conducted by Chengdu Lilai Biological Technology Co., Ltd. (Chengdu, China).

### 4.16. RNA Extraction and RT-qPCR

Real-time quantitative PCR (RT-qPCR) was used to investigate the effects of AEE on the expression of tight junction-related genes. Briefly, total RNA was extracted from the samples using the RNeasy mini kit (TaKaRa, MiniBEST Universal RNA Extraction kit, Code No.9767) according to the manufacturer’s recommendations, and the reverse transcription reaction was carried out using the Superscript kit (TaKaRa, PrimeScriptTMRT reagent Kit with gDNA Eraser, Code No. RR036A). RNA quality and purity were checked spectrophotometrically. RT-qPCR was performed on the ABI ViiATM7 system using the Power SYBR Green PCR Master Mix kit (TaKaRa, TB GreenTM Premix Ex TaqTM, Code No. RR820A). In this study, the relative expression of target genes was calculated using the 2^−ΔΔCt^ method. Primer sequences are shown in Table S2, with β-actin as the internal reference gene.

### 4.17. Metagenome Sequencing for the Contents of the Cecum

At the end of the experiment, the cecum contents were collected under aseptic conditions, and all samples were stored frozen at −80 °C for subsequent analysis. The MagPure Soil DNA KF kit (MGBio) was used to extract total DNA from the cecum contents, and all procedures were based on the manufacturer’s instructions. Samples were delivered to LC Bio Technology Co., Ltd. (Hangzhou, China) for analysis. Library construction and metagenome sequencing were performed by LC Bio Technology Co., Ltd. (Hangzhou, China).

#### 4.17.1. DNA Extractions

DNA from different samples was extracted using CTAB according to the manufacturer’s instructions. The reagent which was designed to uncover DNA from trace amounts of a sample has been shown to be effective for the preparation of the DNA of most bacteria. Sample blanks consisted of unused swabs processed through DNA extraction and were tested to contain no DNA amplicons. The total DNA was eluted in 50 µL of elution buffer by a modification of the procedure described by the manufacturer (Qiagen, Hilden, Germany) and stored at −80 °C until measurement in the PCR by LC-Biotechnologies (Hangzhou) Co., Ltd., Hang Zhou, China.

#### 4.17.2. DNA Library Construction

The DNA library was constructed by the TruSeq Nano DNA LT Library Preparation Kit (FC-121-4001). DNA was fragmented by dsDNA Fragmentase (NEB, M0348S) by incubation at 37 °C for 30 min. Library construction begins with fragmented cDNA. Blunt-end DNA fragments are generated using a combination of fill-in reactions and exonuclease activity, and size selection is performed with provided sample purification beads. An A-base is then added to the blunt ends of each strand, preparing them for ligation to the indexed adapters. Each adapter contains a T-base overhang for ligating the adapter to the A-tailed fragmented DNA. These adapters contain the full complement of sequencing primer hybridization sites for single, paired-end, and indexed reads. Single- or dual-index adapters are ligated to the fragments and the ligated products are amplified with PCR by the following conditions: initial denaturation at 95 °C for 3 min; 8 cycles of denaturation at 98 °C for 15 s, annealing at 60 °C for 15 s, and extension at 72 °C for 30 s; and then final extension at 72 °C for 5 min.

#### 4.17.3. Data Analysis

Raw sequencing reads were processed to obtain valid reads for further analysis. First, sequencing adapters were removed from sequencing reads using cutadapt v1.9. Secondly, low-quality reads were trimmed by fqtrim v0.94 using a sliding-window algorithm. Thirdly, reads were aligned to the host genome using bowtie2 v2.2.0 to remove host contamination. Once quality-filtered reads were obtained, they were de novo assembled to construct the metagenome for each sample by MEGAHIT v1.2.9. All coding regions (CDSs) of metagenomic contigs were predicted by MetaGeneMark v3.26. CDS sequences of all samples were clustered by CD-HIT v4.6.1 to obtain unigenes. The unigene abundance of a certain sample was estimated by TPM based on the number of aligned reads by bowtie2 v2.2.0. The lowest common ancestor taxonomy of unigenes was obtained by aligning them against the NCBI NR database with DIAMOND v 0.9.14.

### 4.18. Statistical Analysis

All data were presented as means ± SD. The differences among different treatment groups were analyzed with one-way ANOVA followed by Duncan’s multiple comparisons and Student’s *t*-test was used for comparison between two groups. Statistical significance was considered at *p* < 0.05.

## 5. Conclusions

In summary, this study showed that AEE could improve the LPS-induced inflammatory response and reduce inflammatory factor levels and oxidative stress levels, thereby improving intestinal barrier damage caused by LPS. The potential molecular mechanisms of AEE include (1) inhibition of NF-κB and MMP-9 activation; (2) increased expression of tight junction-related proteins and genes; and (3) improved flora structure. These findings provide a new theoretical basis for AEE in the treatment of intestinal inflammation.

## Data Availability

The data that support the findings of this study are available from the corresponding author upon reasonable request. Some data may not be made available because of privacy or ethical restrictions.

## Supplementary Materials

The following supporting information can be downloaded at https://www.mdpi.com/article/10.3390/ijms242417434/s1.

## Abbreviations

AEE	Aspirin eugenol ester
LPS	Lipopolysaccharide
AP side	Side of the intestine
BL side	Lower chamber is the basal side
Papp	Apparent permeability coefficient
LY	Lucifer yellow
TEER	Transepithelial electrical resistance
TJ	Tight junction
DAI	Disease Activity Index
ELISA	Enzyme-linked immunosorbent assay
TNF-α	Tumor necrosis factor-α
IL-6	Interleukin-6
IL-1β	Interleukin-1β
LDH	Lactate dehydrogenase
GSH	Glutathione
SOD	Superoxide dismutase
NF-κB	Nuclear factor-kappa B
MMP-9	Matrix metalloproteinase-9
ROS	Reactive oxygen species
COX	Cyclooxygenase
LOX	Lipoxygenase
LTs	Leukotrienes
AA	Arachidonic acid
HE	Hematoxylin–Eosin
CCK-8	Cell counting kit-8
BCA	Bicinchoninic acid
CMC-Na	Carboxymethylcellulose

## References

[1] Zmora N., Levy M., Pevsner-Fishcer M., Elinav E. (2017). Inflammasomes and intestinal inflammation. Mucosal Immunol..

[2] Ash C. (2017). Protection and resolvin gut inflammation. Science.

[3] Du J., Chen Y., Shi Y., Liu T., Cao Y., Tang Y., Ge X., Nie H., Zheng C., Li Y.C. (2015). 1,25-Dihydroxyvitamin D Protects Intestinal Epithelial Barrier by Regulating the Myosin Light Chain Kinase Signaling Pathway. Inflamm. Bowel Dis..

[4] Suzuki T. (2013). Regulation of intestinal epithelial permeability by tight junctions. Cell. Mol. Life Sci..

[5] Hartsock A., Nelson W.J. (2008). Adherens and tight junctions: Structure, function and connections to the actin cytoskeleton. Biochim. Biophys. Acta Biomembr..

[6] Yang G., Bibi S., Du M., Suzuki T., Zhu M.-J. (2017). Regulation of the intestinal tight junction by natural polyphenols: A mechanistic perspective. Crit. Rev. Food Sci. Nutr..

[7] Liu H., Patel N.R., Walter L., Ingersoll S., Sitaraman S.V., Garg P. (2013). Constitutive expression of MMP9 in intestinal epithelium worsens murine acute colitis and is associated with increased levels of proinflammatory cytokine Kc. Am. J. Physiol. Liver Physiol..

[8] Wu X.X., Huang X.L., Chen R.R., Li T., Ye H.J., Xie W., Huang Z.M., Cao G.Z. (2019). Paeoniflorin Prevents Intestinal Barrier Dis-ruption and Inhibits Lipopolysaccharide (LPS)-Induced Inflammation in Caco-2 Cell Monolayers. Inflammation.

[9] Garrett W.S., Gordon J.I., Glimcher L.H. (2010). Homeostasis and Inflammation in the Intestine. Cell.

[10] Schirmer M., Garner A., Vlamakis H., Xavier R.J. (2019). Microbial genes and pathways in inflammatory bowel disease. Nat. Rev. Microbiol..

[11] Gao J., Xu K., Liu H., Liu G., Bai M., Peng C., Li T., Yin Y. (2018). Impact of the Gut Microbiota on Intestinal Immunity Mediated by Tryptophan Metabolism. Front. Cell. Infect. Microbiol..

[12] Ramos G.P., Papadakis K.A. (2019). Mechanisms of Disease: Inflammatory Bowel Diseases. Mayo Clin. Proc..

[13] Kagan J.C. (2017). Lipopolysaccharide Detection across the Kingdoms of Life. Trends Immunol..

[14] Han F., Lu Z., Liu Y., Xia X., Zhang H., Wang X., Wang Y. (2016). Cathelicidin-BF ameliorates lipopolysaccharide-induced intestinal epithelial barrier disruption in rat. Life Sci..

[15] Zhang Z.-D., Huang M.-Z., Yang Y.-J., Liu X.-W., Qin Z., Li S.-H., Li J.-Y. (2020). Aspirin Eugenol Ester Attenuates Paraquat-Induced Hepatotoxicity by Inhibiting Oxidative Stress. Front. Physiol..

[16] Huang M.-Z., Zhang Z.-D., Yang Y.-J., Liu X.-W., Qin Z., Li J.-Y. (2020). Aspirin Eugenol Ester Protects Vascular Endothelium from Oxidative Injury by the Apoptosis Signal Regulating Kinase-1 Pathway. Front. Pharmacol..

[17] Huang M.Z., Yang Y.J., Liu X.W., Qin Z., Li J.Y. (2019). Aspirin Eugenol Ester Reduces H(_2_)O(_2_)-Induced Oxidative Stress of HU-VECs via Mitochondria-Lysosome Axis. Oxid. Med. Cell Longev..

[18] Huang M.Z., Lu X.R., Yang Y.J., Liu X.W., Qin Z., Li J.Y. (2019). Cellular Metabolomics Reveal the Mechanism Underlying the Anti-Atherosclerotic Effects of Aspirin Eugenol Ester on Vascular Endothelial Dysfunction. Int. J. Mol. Sci..

[19] Huang M., Yang Y., Liu X., Qin Z., Li J. (2019). Aspirin eugenol ester attenuates oxidative injury of vascular endothelial cells by regulating NOS and Nrf2 signalling pathways. Br. J. Pharmacol..

[20] Rubin D.T., Ananthakrishnan A.N., Siegel C.A., Sauer B.G., Long M.D. (2019). ACG Clinical Guideline: Ulcerative Colitis in Adults. Am. J. Gastroenterol..

[21] Huang L., Zheng J., Sun G., Yang H., Sun X., Yao X., Lin A., Liu H. (2022). 5-Aminosalicylic acid ameliorates dextran sulfate sodium-induced colitis in mice by modulating gut microbiota and bile acid metabolism. Cell. Mol. Life Sci. CMLS.

[22] Sartor R.B. (2006). Mechanisms of disease: Pathogenesis of Crohn’s disease and ulcerative colitis. Nature clinical practice. Gastroenterol. Hepatol..

[23] de Barboza G.D., Guizzardi S., Moine L., Tolosa de Talamoni N. (2017). Oxidative stress, antioxidants and intestinal calcium absorption. World J. Gastroenterol..

[24] Tao Q., Zhang Z.D., Qin Z., Liu X.W., Li S.H., Bai L.X., Ge W.B., Li J.Y., Yang Y.J. (2022). Aspirin eugenol ester alleviates lipopolysaccharide-induced acute lung injury in rats while stabilizing serum metabolites levels. Front. Immunol..

[25] Zhang Z.D., Yang Y.J., Qin Z., Liu X.W., Li S.H., Bai L.X., Li J.Y. (2021). Protective Activity of Aspirin Eugenol Ester on Paraquat-Induced Cell Damage in SH-SY5Y Cells. Oxid. Med. Cell Longev..

[26] Zhang M., Zhao Y., Yao Y., Xu M., Du H., Wu N., Tu Y. (2019). Isolation and identification of peptides from simulated gastrointestinal digestion of preserved egg white and their anti-inflammatory activity in TNF-α-induced Caco-2 cells. J. Nutr. Biochem..

[27] Bodega G., Alique M., Puebla L., Carracedo J., Ramírez R.M. (2019). Microvesicles: ROS scavengers and ROS producers. J. Extracell. Vesicles.

[28] Coskun M., Vermeire S., Nielsen O.H. (2017). Novel Targeted Therapies for Inflammatory Bowel Disease. Trends Pharmacol. Sci..

[29] Nguyen H.T., Vu T.-Y., Chandi V., Polimati H., Tatipamula V.B. (2020). Dual COX and 5-LOX inhibition by clerodane diterpenes from seeds of *Polyalthia longifolia* (Sonn.) Thwaites. Sci. Rep..

[30] Tanaka K., Suemasu S., Ishihara T., Tasaka Y., Arai Y., Mizushima T. (2009). Inhibition of both COX-1 and COX-2 and resulting decrease in the level of prostaglandins E2 is responsible for non-steroidal anti-inflammatory drug (NSAID)-dependent exacerbation of colitis. Eur. J. Pharmacol..

[31] Tran H.T.T., Márton M.-R., Herz C., Maul R., Baldermann S., Schreiner M., Lamy E. (2016). Nasturtium (Indian cress, Tropaeolum majus nanum) dually blocks the COX and LOX pathway in primary human immune cells. Phytomedicine.

[32] Zoccal K.F., Sorgi C.A., Hori J.I., Paula-Silva F.W.G., Arantes E.C., Serezani C.H., Zamboni D.S., Faccioli L.H. (2016). Opposing roles of LTB4 and PGE2 in regulating the inflammasome-dependent scorpion venom-induced mortality. Nat. Commun..

[33] Tu J., Xu Y., Xu J., Ling Y., Cai Y. (2016). Chitosan nanoparticles reduce LPS-induced inflammatory reaction via inhibition of NF-κB pathway in Caco-2 cells. Int. J. Biol. Macromol..

[34] Yuan J., Che S., Ruan Z., Song L., Tang R., Zhang L. (2021). Regulatory effects of flavonoids luteolin on BDE-209-induced intestinal epithelial barrier damage in Caco-2 cell monolayer model. Food Chem. Toxicol..

[35] Li Y.C., Chen Y., Du J. (2015). Critical roles of intestinal epithelial vitamin D receptor signaling in controlling gut mucosal inflammation. J. Steroid Biochem. Mol. Biol..

[36] Tsukita S., Tanaka H., Tamura A. (2019). The Claudins: From Tight Junctions to Biological Systems. Trends Biochem. Sci..

[37] Bhat A.A., Uppada S., Achkar I.W., Hashem S., Yadav S.K., Shanmugakonar M., Al-Naemi H.A., Haris M., Uddin S. (2018). Tight Junction Proteins and Signaling Pathways in Cancer and Inflammation: A Functional Crosstalk. Front. Physiol..

[38] McGuckin M.A., Eri R., Simms L.A., Florin T.H., Radford-Smith G. (2009). Intestinal barrier dysfunction in inflammatory bowel diseases. Inflamm. Bowel Dis..

[39] Turner J.R. (2009). Intestinal mucosal barrier function in health and disease. Nat. Rev. Immunol..

[40] Zhou X., Wu Y., Ye L., Wang Y., Zhang K., Wang L., Huang Y., Wang L., Xian S., Zhang Y. (2019). Aspirin alleviates endothelial gap junction dysfunction through inhibition of NLRP3 inflammasome activation in LPS-induced vascular injury. Acta Pharm. Sin. B.

[41] Hui Q., Ammeter E., Liu S., Yang R., Lu P., Lahaye L., Yang C. (2020). Eugenol attenuates inflammatory response and enhances barrier function during lipopolysaccharide-induced inflammation in the porcine intestinal epithelial cells. J. Anim. Sci..

[42] Srinivasan B., Kolli A.R., Esch M.B., Abaci H.E., Shuler M.L., Hickman J.J. (2015). TEER measurement techniques for in vitro barrier model systems. J. Lab. Autom..

[43] Chen L., Lu X., Liang X., Hong D., Guan Z., Guan Y., Zhu W. (2016). Mechanistic studies of the transport of peimine in the Caco-2 cell model. Acta Pharm. Sin. B.

[44] Jiao N., Wu Z., Ji Y., Wang B., Dai Z., Wu G. (2015). L-Glutamate Enhances Barrier and Antioxidative Functions in Intestinal Porcine Epithelial Cells. J. Nutr..

[45] Yue Y., Guo Y., Yang Y. (2017). Effects of dietary l-tryptophan supplementation on intestinal response to chronic unpredictable stress in broilers. Amino Acids.

[46] Chen Q., Chen O., Martins I.M., Hou H., Zhao X., Blumberg J.B., Li B. (2017). Collagen peptides ameliorate intestinal epithelial barrier dysfunction in immunostimulatory Caco-2 cell monolayers via enhancing tight junctions. Food Funct..

[47] Chen M., Liu Y., Xiong S., Wu M., Li B., Ruan Z., Hu X. (2019). Dietary l-tryptophan alleviated LPS-induced intestinal barrier injury by regulating tight junctions in a Caco-2 cell monolayer model. Food Funct..

[48] Qiao R., Sheng C., Lu Y., Zhang Y., Ren H., Lemos B. (2019). Microplastics induce intestinal inflammation, oxidative stress, and disorders of metabolome and microbiome in zebrafish. Sci. Total Environ..

[49] Wei X., Li N., Wu X., Cao G., Qiao H., Wang J., Hao R. (2023). The preventive effect of Glycyrrhiza polysaccharide on lipopolysaccharide-induced acute colitis in mice by modulating gut microbial communities. Int. J. Biol. Macromol..

[50] Jobin C., Sartor R.B. (2000). The I kappa B/NF-kappa B system: A key determinant of mucosalinflammation and protection. Am. J. Physiol. Cell Physiol..

[51] Perkins N.D. (2007). Integrating cell-signalling pathways with NF-kappaB and IKK function. Nat. Rev. Mol. Cell Biol..

[52] Raish M., Ahmad A., Ansari M.A., Alkharfy K.M., Aljenoobi F.I., Jan B.L., Al-Mohizea A.M., Khan A., Ali N. (2018). Momordica charantia polysaccharides ameliorate oxidative stress, inflammation, and apoptosis in ethanol-induced gastritis in mucosa through NF-kB signaling pathway inhibition. Int. J. Biol. Macromol..

[53] Nunes C., Almeida L., Barbosa R.M., Laranjinha J. (2017). Luteolin suppresses the JAK/STAT pathway in a cellular model of intestinal inflammation. Food Funct..

[54] Nunes C., Freitas V., Almeida L., Laranjinha J. (2019). Red wine extract preserves tight junctions in intestinal epithelial cells under inflammatory conditions: Implications for intestinal inflammation. Food Funct..

[55] de Bruyn M., Vandooren J., Ugarte-Berzal E., Arijs I., Vermeire S., Opdenakker G. (2016). The molecular biology of matrix metalloproteinases and tissue inhibitors of metalloproteinases in inflammatory bowel diseases. Crit. Rev. Biochem. Mol. Biol..

[56] Li W., Gao M., Han T. (2020). *Lycium barbarum* polysaccharides ameliorate intestinal barrier dysfunction and inflammation through the MLCK-MLC signaling pathway in Caco-2 cells. Food Funct..

[57] Guo W., Zeng M., Zhu S., Li S., Qian Y., Wu H. (2022). Phycocyanin ameliorates mouse colitis via phycocyanobilin-dependent antioxidant and anti-inflammatory protection of the intestinal epithelial barrier. Food Funct..

[58] de Bruyn M., Sabino J., Vandeputte D., Vermeire S., Raes J., Opdenakker G. (2018). Comparisons of gut microbiota profiles in wild-type and gelatinase B/matrix metalloproteinase-9-deficient mice in acute DSS-induced colitis. Npj Biofilms Microbiomes.

[59] de Bruyn M., Breynaert C., Arijs I., De Hertogh G., Geboes K., Thijs G., Matteoli G., Hu J., Van Damme J., Arnold B. (2017). Inhibition of gelatinase B/MMP-9 does not attenuate colitis in murine models of inflammatory bowel disease. Nat. Commun..

[60] Bouchet S., Bauvois B. (2014). Neutrophil Gelatinase-Associated Lipocalin (NGAL), Pro-Matrix Metalloproteinase-9 (pro-MMP-9) and Their Complex Pro-MMP-9/NGAL in Leukaemias. Cancers.

[61] Bchir S., Nasr H.B., Bouchet S., Benzarti M., Garrouch A., Tabka Z., Susin S., Chahed K., Bauvois B. (2017). Concomitant elevations of MMP-9, NGAL, proMMP-9/NGAL and neutrophil elastase in serum of smokers with chronic obstructive pulmonary disease. J. Cell. Mol. Med..

[62] Yu Z., Li D., Sun H. (2023). Herba Origani alleviated DSS-induced ulcerative colitis in mice through remolding gut microbiota to regulate bile acid and short-chain fatty acid metabolisms. Biomed. Pharmacother..

[63] Cao Y., Teng Y., Liu H., Li J., Zhu B., Xia X. (2023). Rhopilema esculentum polysaccharides enhance epithelial cell barrier in vitro and alleviate chronic colitis in mice. Int. J. Biol. Macromol..

[64] Liu S., Zhao W., Lan P., Mou X. (2020). The microbiome in inflammatory bowel diseases: From pathogenesis to therapy. Protein Cell.

[65] Ni J., Wu G.D., Albenberg L., Tomov V.T. (2017). Gut microbiota and IBD: Causation or correlation?. Nat. Rev. Gastroenterol. Hepatol..

[66] Rinninella E., Raoul P., Cintoni M., Franceschi F., Miggiano G.A.D., Gasbarrini A., Mele M.C. (2019). What is the Healthy Gut Microbiota Composition? A Changing Ecosystem across Age, Environment, Diet, and Diseases. Microorganisms.

[67] Xu D., Wu Q., Liu W., Hu G., Meng H., Wang J. (2023). Therapeutic efficacy and underlying mechanisms of Gastrodia elata polysaccharides on dextran sulfate sodium-induced inflammatory bowel disease in mice: Modulation of the gut microbiota and improvement of metabolic disorders. Int. J. Biol. Macromol..

[68] Yang M., Gu Y., Li L., Liu T., Song X., Sun Y., Cao X., Wang B., Jiang K., Cao H. (2021). Bile Acid-Gut Microbiota Axis in Inflammatory Bowel Disease: From Bench to Bedside. Nutrients.

[69] Chiang J.Y. (2013). Bile acid metabolism and signaling. Compr. Physiol..

[70] Jia W., Xie G., Jia W. (2018). Bile acid-microbiota crosstalk in gastrointestinal inflammation and carcinogenesis. Nat. Rev. Gastroenterol. Hepatol..

[71] Xiao-Rong L., Ning M., Xi-Wang L., Shi-Hong L., Zhe Q., Li-Xia B., Ya-Jun Y., Jian-Yong L. (2021). Untargeted and Targeted Metabolomics Reveal the Underlying Mechanism of Aspirin Eugenol Ester Ameliorating Rat Hyperlipidemia via Inhibiting FXR to Induce CYP7A1. Front. Pharmacol..

[72] Li H., Gong Y., Xie Y., Sun Q., Li Y. (2018). Clostridium butyricum protects the epithelial barrier by maintaining tight junction protein expression and regulating microflora in a murine model of dextran sodium sulfate-induced colitis. Scand. J. Gastroenterol..

[73] Huang B., Wang L., Liu M., Wu X., Lu Q., Liu R. (2022). The underlying mechanism of A-type procyanidins from peanut skin on DSS -induced ulcerative colitis mice by regulating gut microbiota and metabolism. J. Food Biochem..

[74] Iljazovic A., Roy U., Gálvez E.J.C., Lesker T.R., Zhao B., Gronow A., Amend L., Will S.E., Hofmann J.D., Pils M.C. (2021). Perturbation of the gut microbiome by *Prevotella* spp. enhances host susceptibility to mucosal inflammation. Mucosal Immunol..

[75] Liu X., Zhang Y., Li W., Zhang B., Yin J., Liuqi S., Wang J., Peng B., Wang S. (2022). Fucoidan Ameliorated Dextran Sulfate Sodium-Induced Ulcerative Colitis by Modulating Gut Microbiota and Bile Acid Metabolism. J. Agric. Food Chem..

