# Supplementary material for: Protective Effect and Mechanism of Aspirin Eugenol Ester on Lipopolysaccharide-Induced Intestinal Barrier Injury
Source: Int J Mol Sci. 2023 Dec 13;24(24):17434. doi: 10.3390/ijms242417434 (PMC10743450; doi:10.3390/ijms242417434)
Supplement: Supplementary file 1 [file ijms-24-17434-s001.zip › ijms-2719744-supplementary.pdf]

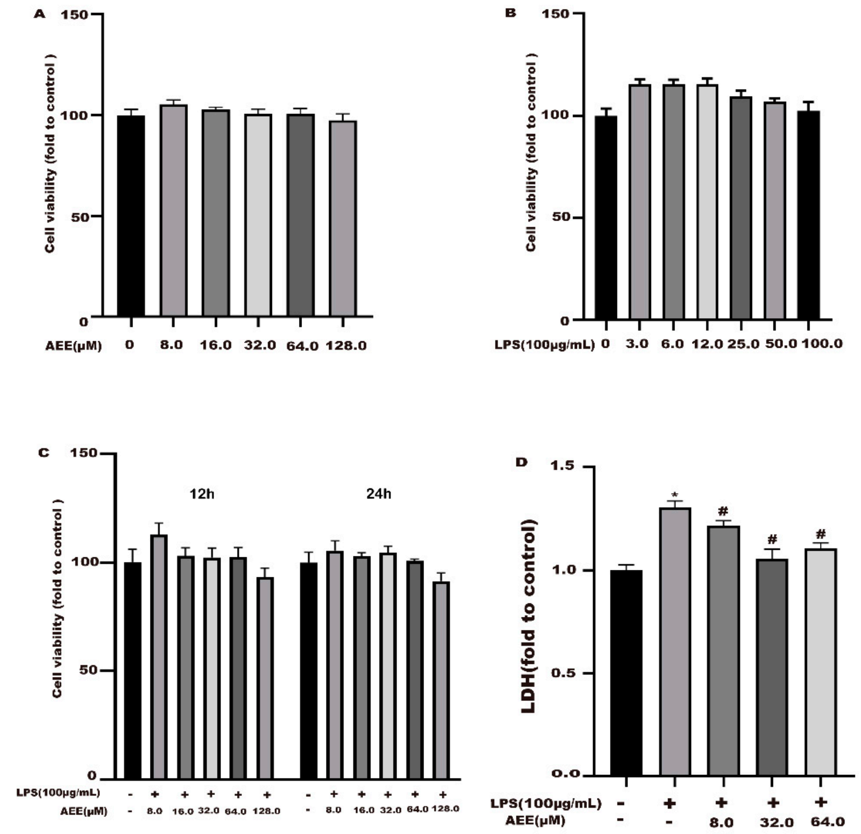

Figure S1 Effect of AEE on LPS-induced cytotoxicity in Caco-2 cells. (A) Cell viability of Caco-2 cells treated with AEE (0 -128 μM) for 24 h. (B) Cell viability of Caco-2 cells treated with LPS (0 - 100 μg/mL) for 24 h. (C) Cell viability of Caco-2 cells pretreated with AEE (8, 32, 64 μM) for 24 h and then treated with LPS (100 μg/mL) for 12 h and 24 h. (D) LDH leakage of Caco-2 cells pretreated with AEE (8, 32, 64 μM) for 24h and then treated with LPS (100 μg/mL) for 24h. Values are presented as the means ± SD where applicable (n = 6). $^{*}P < 0.05$ compared to control group, $^{\#}P < 0.05$ compared to LPS group.

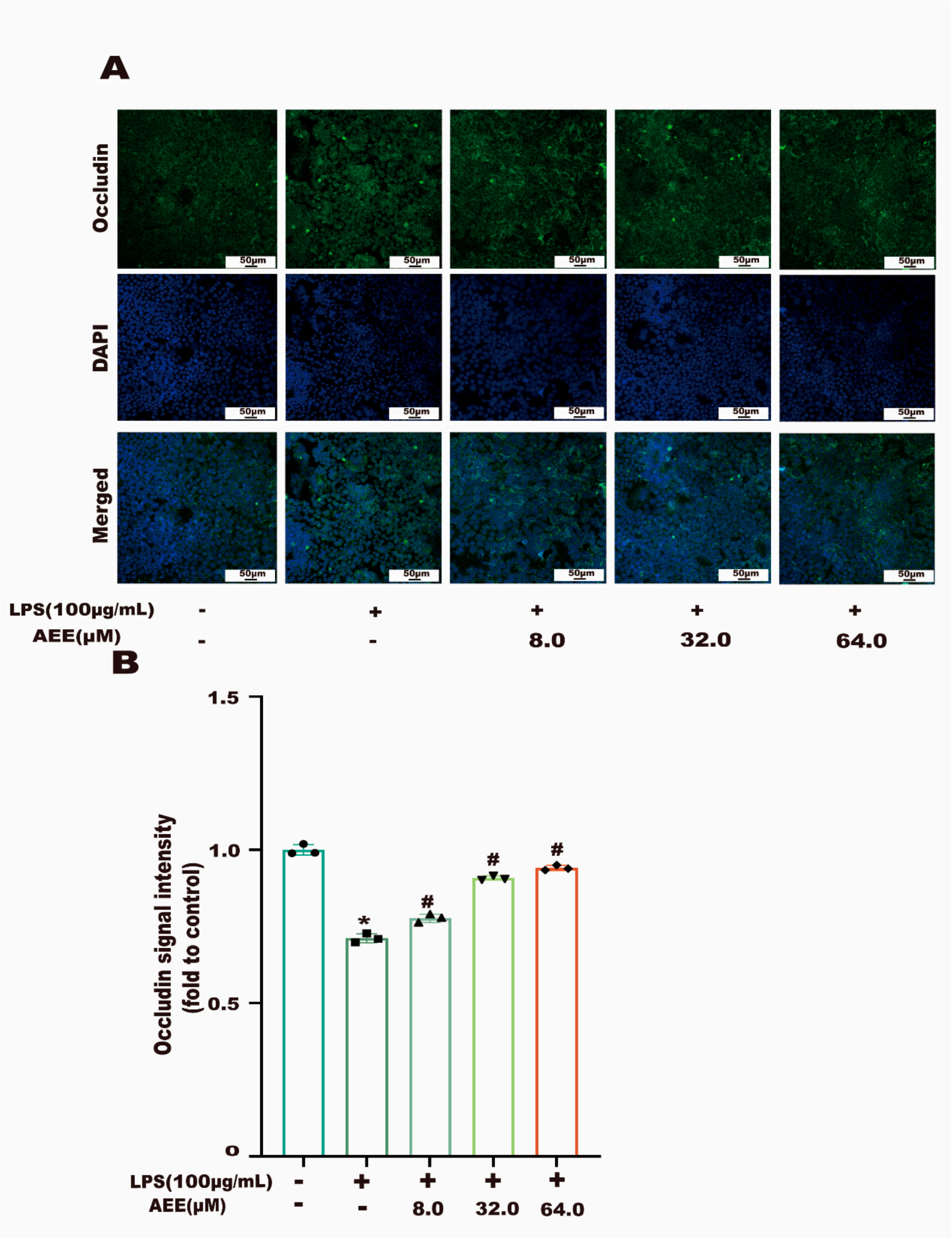

Figure S2 Effect of AEE on LPS-induced down-regulation of TJ proteins. Caco-2 cells were pretreated with AEE (8, 32, 64 μM) for 24 h and then treated with LPS (100 μg·mL^{-1}) for 24 h. (A) Immunofluorescence staining of TJ proteins Occludin in Caco-2 cells. (B) Relative fluorescence intensity of Occludin was calculated by Image J software1.8.0. Values are presented

as the means ± SD where applicable ($n = 3$). $^{*}P < 0.05$ compared to control group, $^{\#}P < 0.05$ compared to LPS group. One-way ANOVA followed with Duncan's multiple comparisons.

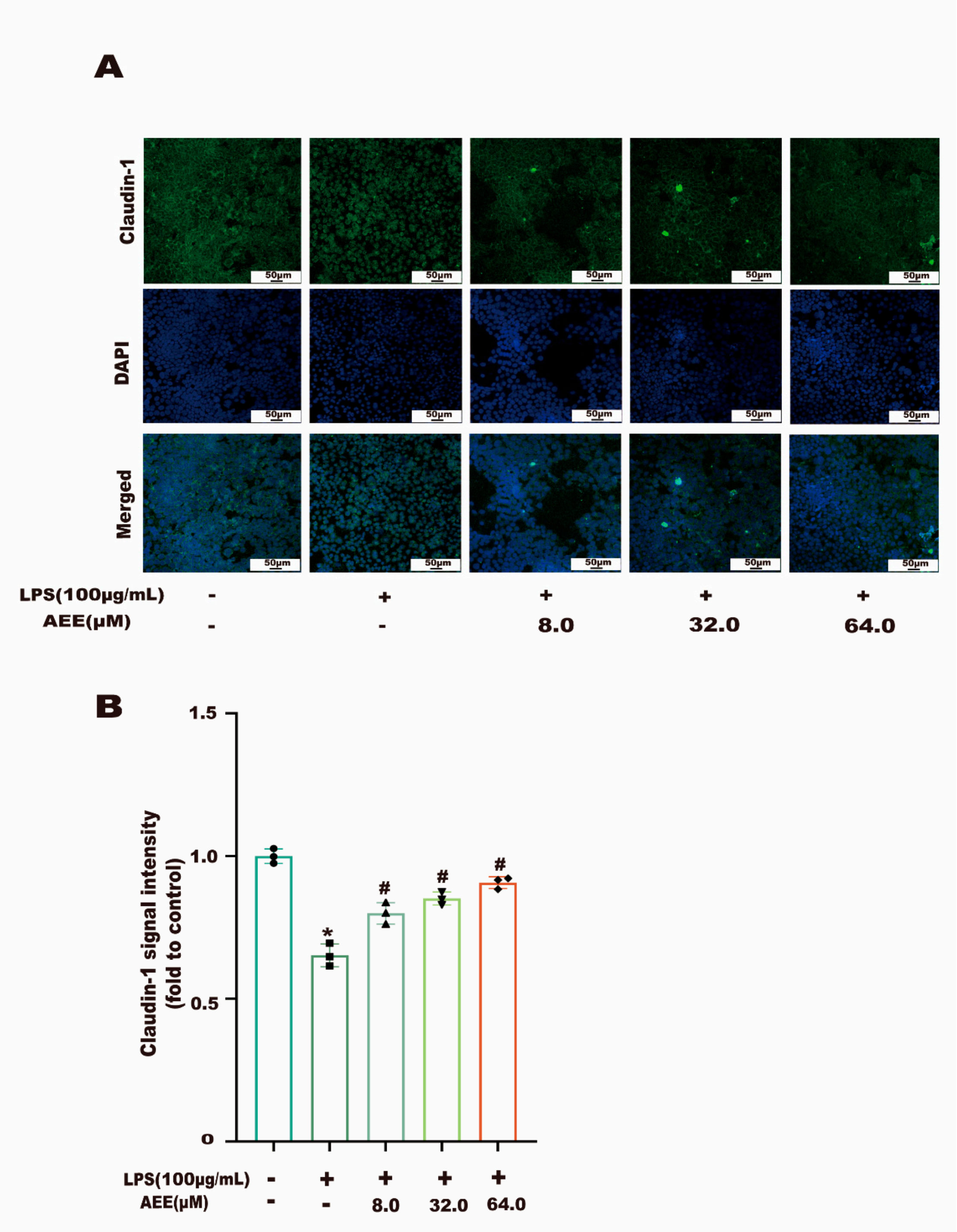

Figure S3 Effect of AEE on LPS-induced down-regulation of TJ proteins. Caco-2 cells were pretreated with AEE (8, 32, 64 μM) for 24 h and then treated with LPS (100 μg·mL^{-1}) for 24 h. (A) Immunofluorescence staining of TJ proteins Claudin-1 in Caco-2 cells. (B) Relative fluorescence intensity of Claudin-1 was calculated by Image J software 1.8.0. Values are

presented as the means ± SD where applicable ($n = 3$). $^{*}P < 0.05$ compared to control group, $^{\#}P < 0.05$ compared to LPS group. One-way ANOVA followed with Duncan's multiple comparisons.

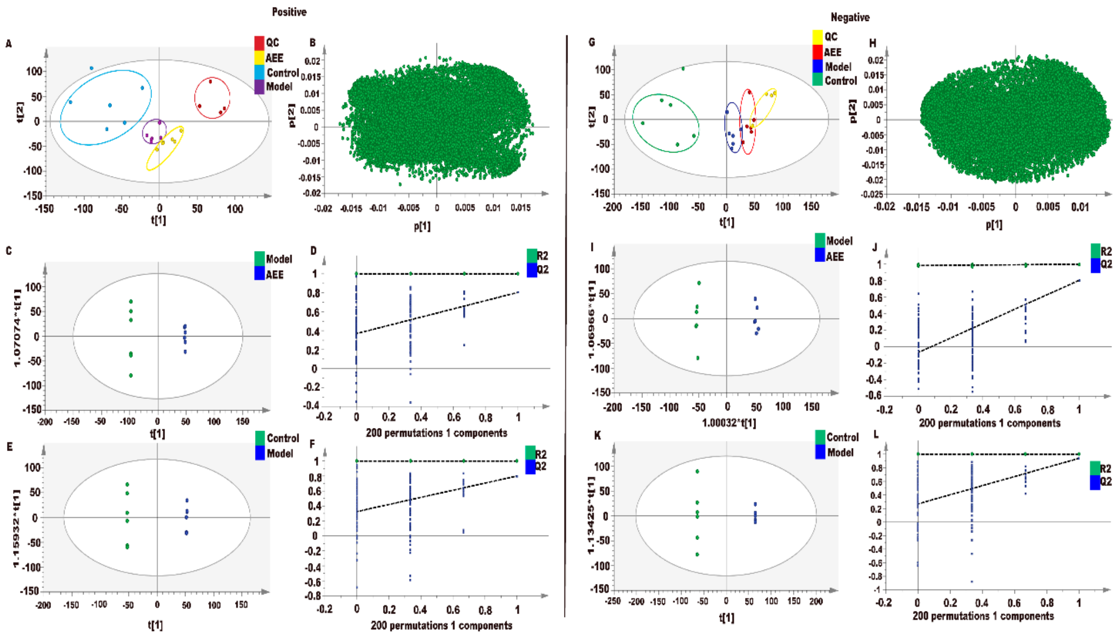

Figure S4 Metabolomic analysis of the effect of AEE on LPS-induced intestinal barrier injury in Caco-2 cells. (A, G) PCA score plots based on Caco-2 cell lysis solution obtained from Control, LPS and AEE groups in ESI^+ and ESI^- modes. (B,H) The loading plots. (C, I) OPLS-DA score plots for AEE and LPS groups in ESI^+ and ESI^- models. (D, J) Permutation test for the OPLS-DA model in ESI^+ and ESI^- modes. (E, K) OPLS-DA score plots for Control and LPS groups in ESI^+ and ESI^- models. (F, L) Permutation test for the OPLS-DA model in ESI^+ and ESI^- modes. Values are presented as the means ± SD where applicable (n = 6).

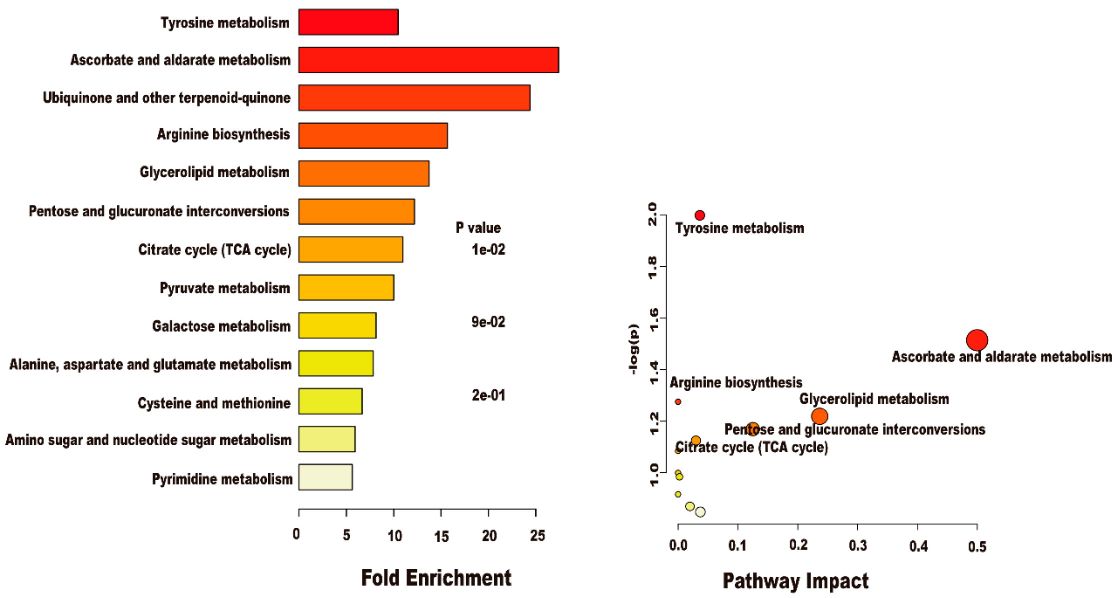

Figure S5 Results of ploidy enrichment and pathway analysis of potential metabolites in Caco-2 cell.

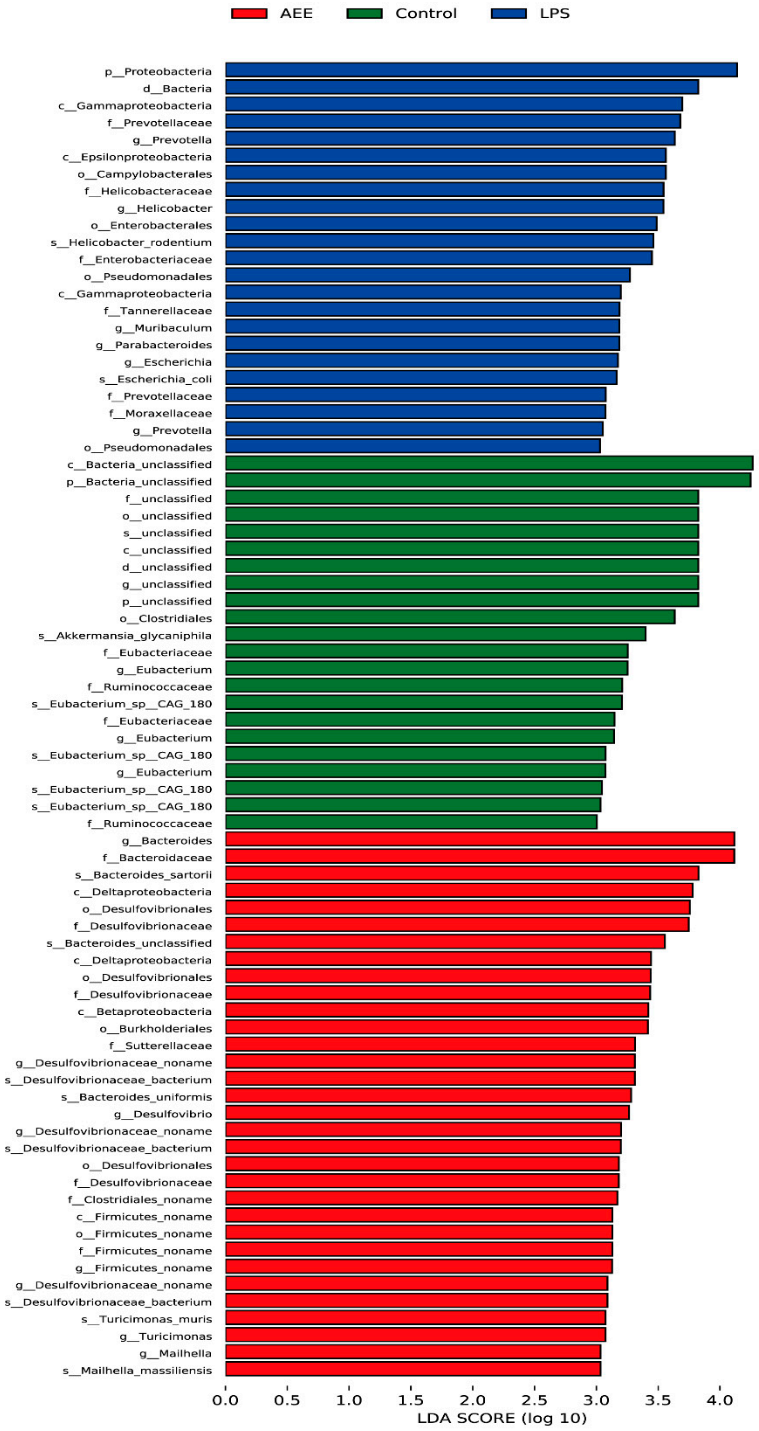

Figure S6 Distribution histogram of differential bacteria based on LDA Score.

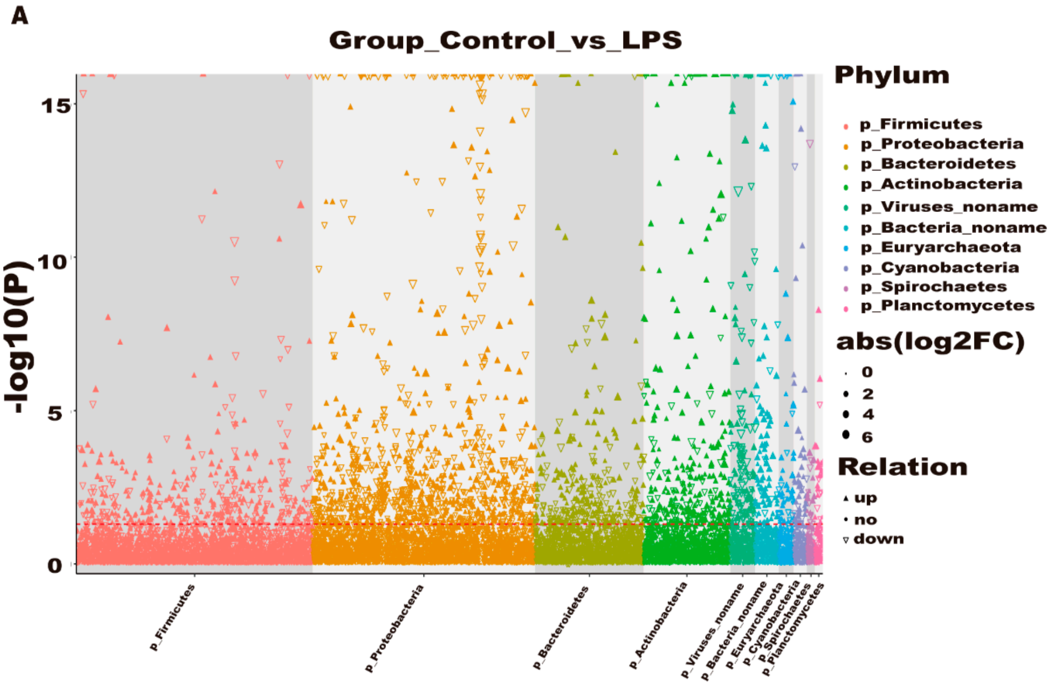

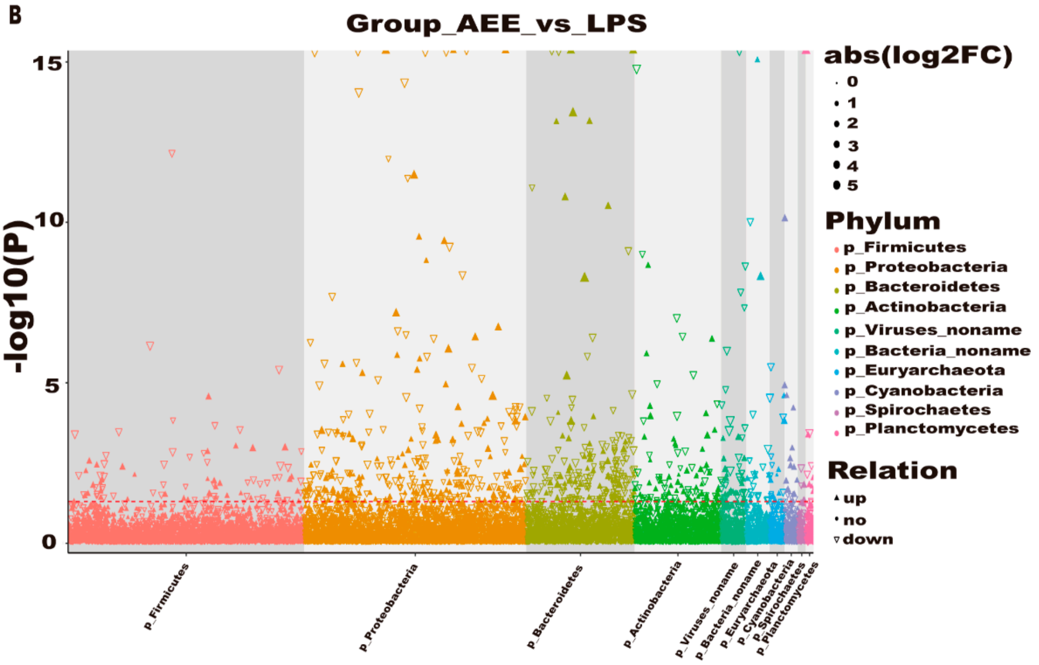

Figure S7 Manhattan plot analysis of abundance variability in samples.

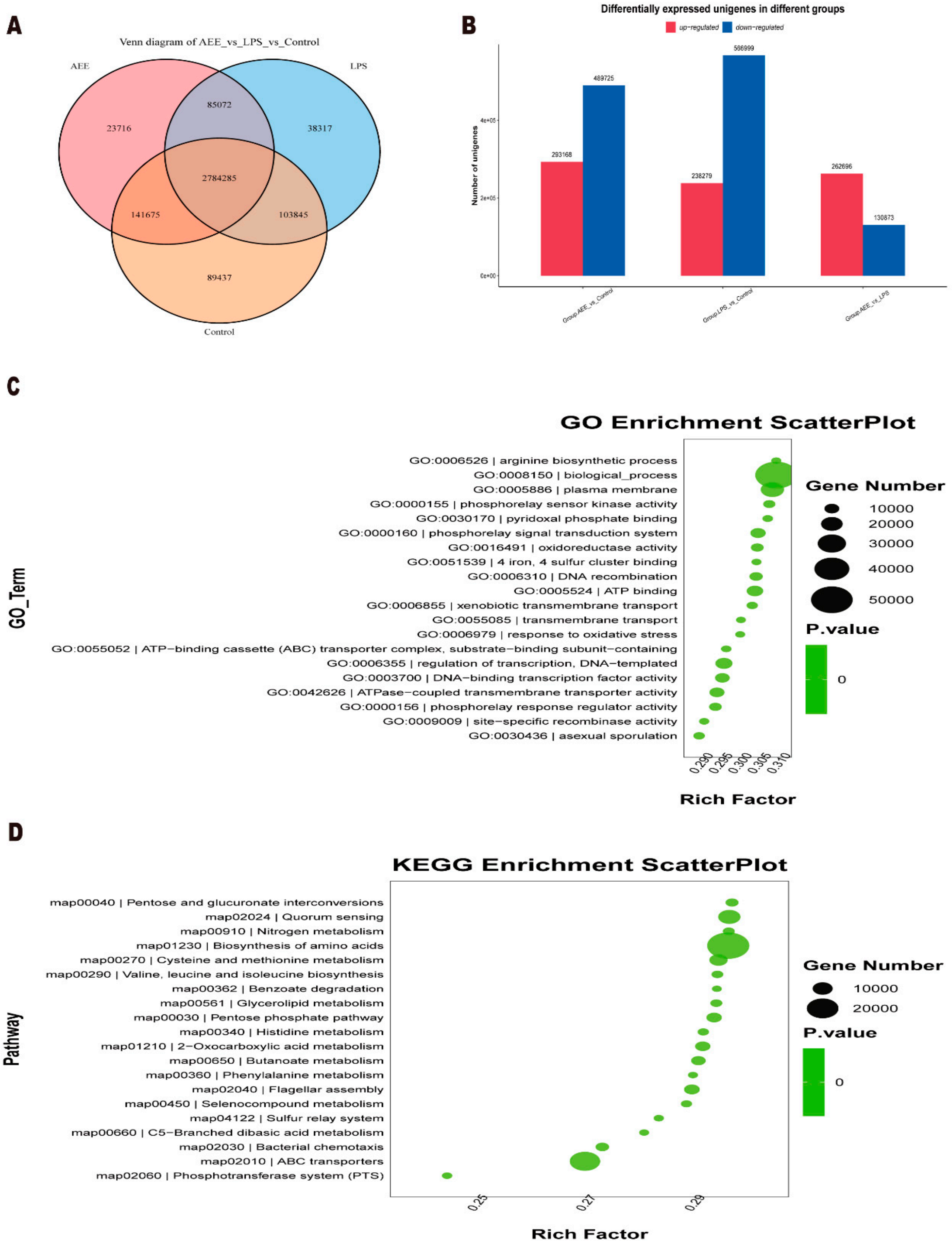

Figure S8 Effects of AEE on the genes in the cecum microbiota. (A) Venn diagram of DEGs. (B) Histogram of DEGs statistics. (C) GO enrichment analysis of DEGs. (D) KEGG pathway analysis of DEGs.

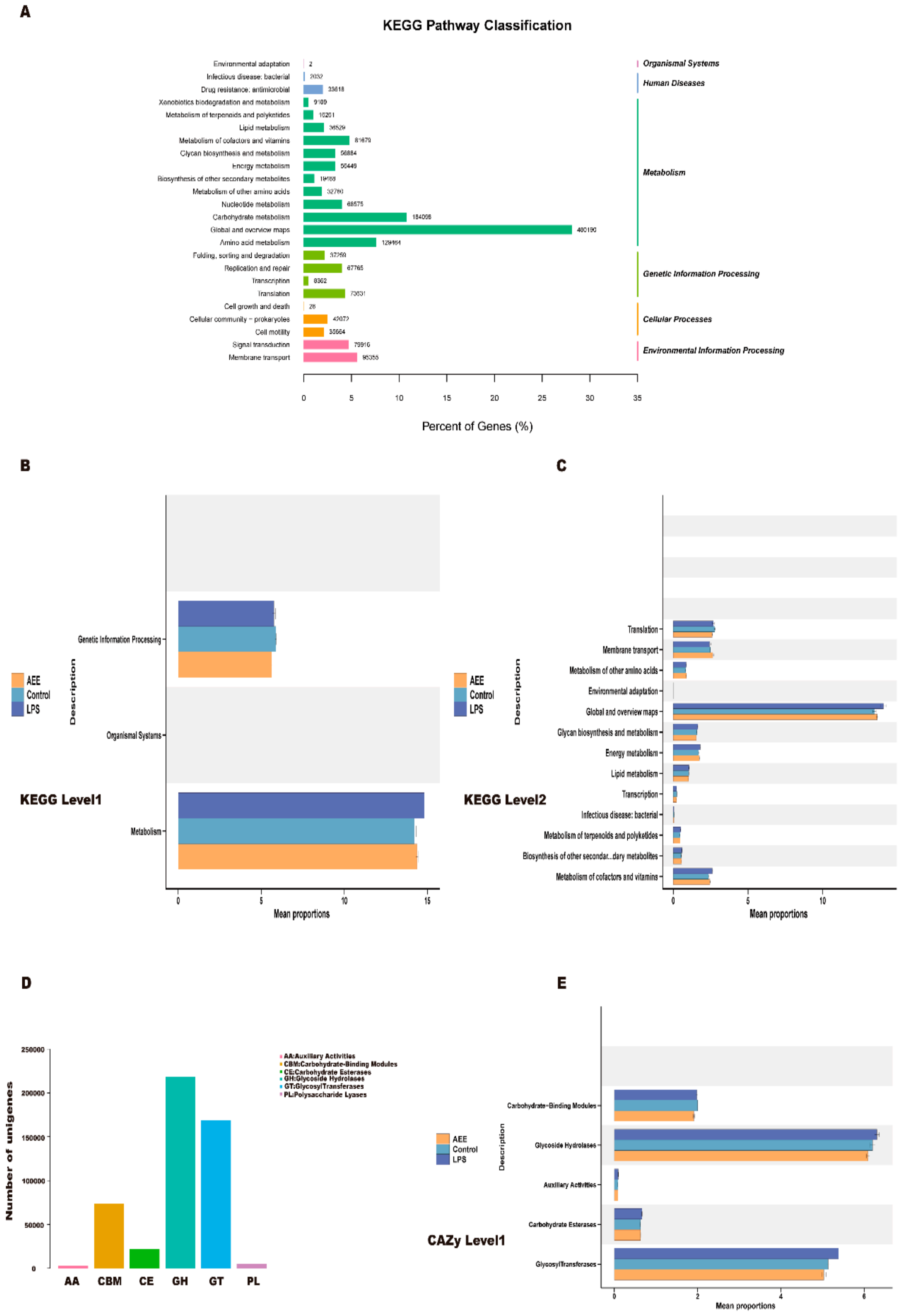

A
KEGG Pathway Classification
Environmental adaptation
Infectious disease: bacterial
Drug resistance: antimicrobial
Xenobiotics biodegradation and metabolism
Metabolism of terpenoids and polyketides
Lipid metabolism
Metabolism of cofactors and vitamins
Glycan biosynthesis and metabolism
Energy metabolism
Biosynthesis of other secondary metabolites
Metabolism of other amino acids
Nucleotide metabolism
Carbohydrate metabolism
Global and overview maps
Amino acid metabolism
Folding, sorting and degradation
Replication and repair
Transcription
Translation
Cell growth and death
Cellular community − prokaryotes
Cell motility
Signal transduction
Membrane transport
Organismal Systems
Human Diseases
Metabolism
Genetic Information Processing
Cellular Processes
Environmental Information Processing
Percent of Genes (%)
B
AEE
Control
LPS
Description
Genetic Information Processing
Organismal Systems
Metabolism
KEGG Level1
Mean proportions
C
AEE
Control
LPS
Description
Translation
Membrane transport
Metabolism of other amino acids
Environmental adaptation
Global and overview maps
Glycan biosynthesis and metabolism
Energy metabolism
Lipid metabolism
Transcription
Infectious disease: bacterial
Metabolism of terpenoids and polyketides
Biosynthesis of other secondar...dary metabolites
Metabolism of cofactors and vitamins
KEGG Level2
Mean proportions
D
Number of unigenes
AA:Auxiliary Activities
CBM:Carbohydrate-Binding Modules
CE:Carbohydrate Esterases
GH:Glycoside Hydrolases
GT:GlycosylTransferases
PL:Polysaccharide Lyases
AA
CBM
CE
GH
GT
PL
E
AEE
Control
LPS
Description
Carbohydrate−Binding Modules
Glycoside Hydrolases
Auxiliary Activities
Carbohydrate Esterases
GlycosylTransferases
CAZy Level1
Mean proportions

Figure S9 Function analysis of DEGs. (A) KEGG pathway analysis. (B) KEGG Level 1 pathway analysis. (C) KEGG Level 2 pathway analysis. (D) CAZy analysis. (E) CAZy Level 1 pathway analysis.

Table S1. The scoring criteria of disease activity index (DAI).

Score	Weight loss	Stool consistency	Blood in the stool
0	None	Normal	Normal
1	≥1 – <5%		
2	≥5 – <10%	Very soft	Slight bleeding
3	≥10 – <20%		
4	≥20%	Watery diarrhea	Gross bleeding

Table S2 Primers used for gene expression analysis.

Gene	Forward Primer(5' → 3')	Reverse Primer(3' → 5')
ZO-1	GCCAAGCCAGTCCATTCTCAGAG	TCCATAGCATCAGTTTCGGGTTTCC
Occludin	CAACGGCAAAGTGAATGGCAAGAG	TCATCCACGGACAAGGTCAGAGG
claudin-1	GCACAGCACTTTACAAGCAACCC	CACCTCGTCATCTTCCAAGCACTTC
NF-κB	GGATGGCTTCTATGAGGCTGAACTC	CTTGCTCCAGGTCTCGCTTCTTC
MMP-9	CACCGCCAACTATGACCAGGATAAG	CTGCTTGCCCAGGAAGACGAAG
β-actin	GCTGTGCTATGTTGCCCTAGACTTC	GGAACCGCTCATTGCCGATAGTG

Table S3. Elution procedure for ultra-high performance liquid chromatography (UPLC) of cell lysate samples.

Samples	Time(min)	A%	B%
	0	95	5
	2	95	5
	12	50	50
Cell lysate	15	5	95
	17	5	95
	18	95	5
	20	95	5

Table S4 statistics of differential metabolites in serum of rats.

No	RT	VIP	Formula	Metabolites	SM	m/z	Fold Change	
							C/LPS	AEE/LPS
1	0.779	1.54232	$C_{11}H_8ClNO_2$	Quinmerac	ESI^+	222.0283	1.13948	0.911347*
2	1.181	1.43928	$C_{16}H_{15}NO_4$	Arborinine	ESI^+	318.99738	1.02742	1.38821*
3	1.935	1.76928	$C_9H_{14}N_2O_{12}P_2$	Uridine 5 -(trihydrogen diphosphate)	ESI^+	405.00934	0.116473	0.384162*
4	5.832	1.62956	$C_{26}H_{43}NO_5$	Glutamate conjugated chenodeoxycholic acid	ESI^+	504.33542	0.0293798	1.22327*
5	7.922	1.5818	$C_{20}H_{20}O_7$	Quercetin pentamethyl ether	ESI^+	373.2926	0.484102	0.899874*
6	8.017	1.63779	$C_{27}H_{39}NO_3$	Jervine	ESI^+	426.29898	0.254385	0.703128*
7	10.13328	1.58396	$C_{24}H_{37}NO_2$	Buxtamine	ESI^+	372.289	0.417552	0.767431*
8	11.78754	1.58805	$C_{27}H_{43}NO_3$	Peiminine	ESI^+	430.3313	0.293559	0.81643
9	12.18932	1.88542	$C_5H_6N_2O_2$	Thymine	ESI^+	127.05	1.04414	0.43148*
10	12.53289	1.65457	$C_6H_{10}ClNO_2$	Isoguvacine	ESI^+	128.0703	2.08319	1.38713*
11	14.07886	1.13421	$C_{14}H_{20}NO_4$	Caffeoylcholine	ESI^+	266.1382	1.47396	0.294387*
12	16.65502	1.60905	$C_4H_4O_4$	Fumaric acid	ESI^+	116.07	0.242712	0.584031*
13	21.9458	1.53487	$C_7H_6O_3$	Salicyclic acid	ESI^+	139.0408	0.702458	0.795896*
14	21.92893	1.70666	$C_6H_{12}N_2O_4S_2$	L-cystine	ESI^+	241.0258	0.301585	0.42144
15	0.483	1.10744	$C_{15}H_{22}N_2O_{18}P_2$	UDP-D-Glucuronic acid	ESI^-	579.0274	0.187663	3.87367*
16	0.803	1.90048	$C_9H_{13}N_3O_5$	Cytarabine	ESI^-	242.07927	1.50211	0.119844*
17	8.374907	1.96119	$C_{16}H_{12}O_4$	6-methoxyflavonol	ESI^-	267.0907	0.901815	0.167697*
18	9.570177	1.30745	$C_{11}H_{14}O_3$	Butylparaben	ESI^-	193.0895	2.22403	2.13378
19	12.60327	1.09474	$C_{15}H_{10}O_6$	Fisetin	ESI^-	285.1266	1.22039	0.370507*
20	13.26955	1.88519	$C_{42}H_{82}NO_{10}P$	Phosphatidylserine 18	ESI^-	834.527	0.908461	0.481689*
21	14.2383	1.50531	$C_{20}H_{24}O_3$	Triptophenolide	ESI^-	311.1689	2.53481	1.74363*
22	14.57199	1.51665	$C_{26}H_{45}NO_5S$	Taurolithocholic acid	ESI^-	482.2951	4.81795	0.638207*
23	15.01705	1.37749	$C_{19}H_{24}N_2O_3$	Labetalol	ESI^-	327.1736	3.43075	2.5946*
24	21.91724	1.00744	$C_{19}H_{22}O_3$	Aurapten	ESI^-	297.1527	0.783095	0.49219*

RT, retention time; VIP, variable importance in the projection; SM, scan mode; +, metabolites identified in positive mode; -, metabolites identified in negative mode. Metabolites identified in both positive and negative modes; $*P < 0.05$ compared to LPS group; C/LPS, control group compared with LPS group; AEE/LPS, AEE group compared with LPS group.
